# Electronic Modulation and Built‐in Electric Field Strategies in Heterostructures Together Induce 1T‐Rich MoS_2_ Conversion for Advanced Sodium Storage

**DOI:** 10.1002/advs.202417288

**Published:** 2025-02-10

**Authors:** Hui Peng, Wenxing Miao, Jingtian Zeng, Zihao Wang, Chenhui Yan, Guofu Ma, Ziqiang Lei

**Affiliations:** ^1^ Key Laboratory of Eco‐functional Polymer Materials of the Ministry of Education Key Laboratory of Polymer Materials of Gansu Province College of Chemistry and Chemical Engineering Northwest Normal University Lanzhou 730070 China

**Keywords:** 1T‐MoS_2_, built‐in electric field, electron modulation, heterojunction, sodium‐ion batteries

## Abstract

1T‐MoS_2_ is considered an attractive energy storage material due to its large layer spacing and excellent electrical conductivity. Unfortunately, 1T‐MoS_2_ is difficult to synthesize directly due to the substability, which limits its development and application. Electron‐filling engineering of Mo 4d orbitals is the core idea to induce an efficient conversion of 2H to 1T phase. Based on this theory, a homogeneous CuS@MoS_2_ heterogeneous nanosheet is successfully constructed based on electron‐rich CuS as an electron donor. Both density functional theory (DFT) and X‐ray absorption fine structure analysis (XAFS) illustrate that part of the electrons from Cu at the heterogeneous interface are transferred to Mo, which triggers the reorganization of Mo 4d orbitals and the formation of a strong built‐in electric field at the interface, and induces an irreversible phase transition from 2H to 1T in MoS_2_. Based on its structural features, CuS@MoS_2_ heterogeneous nanosheets have a high first discharge capacity of 725 mAh g^−1^ at 0.1 A g^−1^, excellent rate performance (466.73 mAh g^−1^ at 10 A g^−1^), and long cycle stability (506.03 mAh g^−1^ after 3200 cycles at 5 A g^−1^). This work provides new perspectives for the development of high‐performance sodium storage anode materials based on 1T‐rich MoS_2_.

## Graphical abstract

A homogeneous CuS@MoS_2_ heterogeneous nanosheet is successfully constructed with electron‐rich CuS as electron donor and induced a phase transition from 2H to 1T in MoS_2_ for advanced sodium storage.

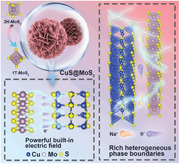

## 1 Introduction

In recent years, with the rising global energy demand and growing concerns about the limitations of traditional lithium‐ion batteries (LIBs), there is an urgent need to develop a clean and efficient new energy storage technology to reduce the reliance on lithium resources in energy storage technology.^[^ ^1^, ^2^, ^3^ ^]^ Among them, sodium‐ion batteries (SIBs) are considered to be the strongest contender to replace LIBs in the second‐generation energy storage boom due to their similar physicochemical properties and operating principles with LIBs, high energy density, high safety, and abundant sodium resources.^[^ ^4^, ^5^, ^6^ ^]^ Unfortunately, the ionic radius of Na^+^ (1.06 Å) is larger than that of Li^+^ (1.02 Å), which results in very slow insertion and de‐insertion of Na^+^ in conventional anode materials and accompanied by severe volume expansion and structural pulverization.^[^ ^7^, ^8^ ^]^ For anode materials, commercial graphite is stable in LIBs, but achieves a specific capacity of only 35 mAh g^−1^ in SIBs.^[^ ^9^, ^10^ ^]^ The hard carbon anode is highly likely to be commercialized but also exhibits only limited sodium storage capacity (200‐300 mAh g^−1^), which still does not meet the demand for high energy density SIBs.^[^ ^9^, ^11^ ^]^ Therefore, it is necessary to develop new high‐performance anode materials to improve the sodium storage capacity and stability.^[^ ^12^, ^13^ ^]^

Transition metal sulfides (M_m_S_n_) are considered as a promising electrochemical energy storage material due to their controllable structure and high capacity based on multistep transformation reactions.^[^ ^14^, ^15^, ^16^, ^17^, ^18^ ^]^ Among them, MoS_2_ has attracted much attention as an ideal anode candidate for SIBs due to its unique layered structure with a large layer spacing (0.62 nm), which can realize high theoretical capacity (670 mAh g^−1^).^[^ ^19^, ^20^, ^21^, ^22^ ^]^ Based on the distinct coordination modes of S and Mo, MoS_2_ can be classified into two phases: the 2H phase (featuring a trigonal prism structure) and the 1T phase (with an octahedral coordination structure). The 2H‐MoS_2_ has intrinsically low conductivity, exhibits semiconducting characteristics, and possesses a relatively large monolayer band gap of 1.9 eV. These properties result in rapid capacity fading and unsatisfactory cycling performance. In contrast, 1T‐MoS_2_ has metallic properties, high conductivity, and larger layer spacing, which provides structural support for fast Na^+^ mass transfer. These advantages make 1T‐MoS_2_ manifest superior performance in the fields of photo‐ and electrocatalysis and energy storage (e.g., in the fields of catalytic hydrogen precipitation, ^[^ ^23^, ^24^ ^]^ energy storage materials,^[^ ^25^ ^]^ and optical materials,^[^ ^26^ ^]^ etc.). Unfortunately, the Mo 4d orbitals of 1T‐MoS_2_ are not completely filled and exhibit low energy levels and substability, which make it difficult to be directly synthesized by conventional synthetic methods, thus greatly limiting its large‐scale applications.^[^ ^21^ ^]^

Currently, researchers have identified various strategies to realize the induced 1T phase transition of MoS_2_ to improve its sodium storage performance, such as ion embedding, atomic interface engineering, and atomic doping. Sun et al. inserted PO_4_^3−^ with a larger ionic radius and higher electronegativity into the 2H‐MoS_2_ interlayer, which led to the transfer of some electrons from PO_4_^3−^ to Mo, triggering the reorganization of Mo 4d orbitals, and realizing the phase transition of MoS_2_ from 2H to 1T, and thus achieving its unparalleled fast‐charging performance.^[^ ^19^ ^]^ In addition to this, Zhang et al. assembled 2H‐MoS_2_ on a monoatomically dispersed Fe‐N‐C (SA Fe‐N‐C) backbone by atomic interface engineering, and the Fe‐S bond acted as an electron transfer channel from SA Fe‐N‐C to 2H‐MoS_2_, which directly regulated the phase transition of MoS_2_ from 2H to 1T during cycling, thus realizing the 1T/2H MoS_2_/SA Fe‐N‐C with excellent sodium storage performance.^[^ ^24^ ^]^ In addition, heteroatom doping promotes the phase transition during MoS_2_ nucleation and growth. Zhao et al. precisely constructed a MoS_2_ multi‐shell hollow structure (Fe‐M‐HoMS) by Fe single‐atom doping strategy, and the strong adsorption and autocatalytic effect of Fe single‐atom on Na^+^ facilitates the reversible conversion between 2H and 1T phases, thus realizing the strong Na^+^ storage capacity and excellent rate performance of Fe‐M‐HoMS.^[^ ^25^ ^]^ As can be seen, the focus of a large number of studies has emphasized that electron transfer from electron donors to Mo achieves equilibrium electron occupation in the Mo 4d orbitals to facilitate the conversion of 1T‐rich MoS_2_. Based on this core idea, the construction of heterostructures of metal sulfides with different bandgap structures can induce spontaneous electron retransfer and redistribution at the heterogeneous interfaces to generate a strong built‐in electric field.^[^ ^20^ ^]^ In this way, the design of a sulfide heterostructure involving MoS_2_ with a sulfide that can act as an electron donor to transfer electrons to it is expected to overcome the above drawbacks, resulting in the realization of stable 1T‐MoS_2_ as well as a strong built‐in electric field for facilitating the transfer of electrons and ions, which has rarely been reported in battery materials.

CuS is a conversion anode material with excellent sodium storage kinetics. Its narrow bandgap and high Fermi energy level endow it with the ability to act as both an electron donor and a good mass transfer carrier.^[^ ^20^, ^29^, ^30^ ^]^ Thanks to this unique property, in this work, we constructed homogeneous CuS@MoS_2_ heterogeneous nanosheets by using a one‐step hydrothermal method in template‐free conditions on molded CuMo‐precursors to guide the uniform growth of bimetallic sulfides during hydrothermal vulcanization. Both density functional theory (DFT) and X‐ray absorption fine structure analysis (XAFS) results illustrate that a part of the electrons from the Cu at the heterointerfaces are transferred to Mo, which triggers a Mo 4d orbital reorganization, inducing an irreversible phase transition from 2H to 1T in MoS_2_. Moreover, the Na^+^ diffusion energy barrier of CuS@MoS_2_ (0.24 eV) is much lower than that of pristine MoS_2_ (0.46 eV) and CuS (0.74 eV). As a result, stabilized CuS@MoS_2_ heterostructure has a high first discharge capacity of 725 mAh g^−1^ at 0.1 A g^−1^ with an ICE of 78%, providing excellent rate performance and long cycle durability. In addition, the full cell assembled from a CuS@MoS_2_ anode and Na_3_V_2_(PO_4_)_3_@C cathode showed good rate performance and long‐term cycling stability, demonstrating its potential for practical applications.

## 2 Result and Discussion

### 2.1 Preparation and Physical Characterization

**Figure 1:**
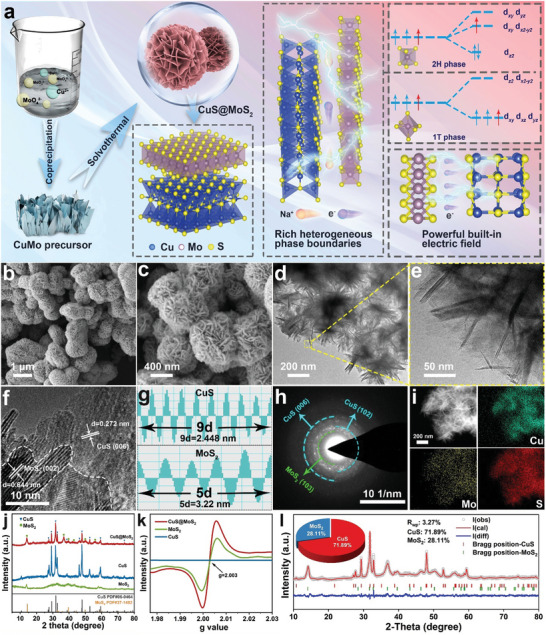
Morphological structure characterizations. a) Preparation process of CuS@MoS_2_ heterostructure. b, c) SEM images, d, e) TEM images, f, g) HRTEM images, h) SAED pattern and i) the corresponding HAADF‐STEM image and EDS elemental mappings of CuS@MoS_2_, j) XRD patterns of CuS@MoS_2_, CuS and MoS_2_, k) EPR spectra of CuS@MoS_2_, CuS, and MoS_2_; k) Refined XRD pattern of CuS@MoS_2_ heterostructure.

**Figure** **1a** demonstrates the preparation process of CuS@MoS_2_ with the heterostructure and built‐in electric field advantages. In line with crystal theory, the Mo 4d orbitals in 2H‐MoS_2_ are split into three energy levels, namely d_z2_, d_x2‐y2_/d_xy_, and d_xz_/d_yz_ orbitals. The electrons fully fill the d_z2_ orbitals of the lower energy levels, endowing 2H‐MoS_2_ with highly stable semiconductor properties. By contrast, the Mo 4d orbital of 1T‐MoS_2_ divides into d_xy_/d_xz_/d_yz_ and d_z2_/d_x2‐y2_ orbitals. As the low‐energy d_xy_/d_xz_/d_yz_ degenerate orbitals aren't fully occupied, during the formation of the CuS@MoS_2_ heterojunction, the extra electrons supplied by CuS, acting as an electron‐rich substance for MoS_2_, initially populate the d_x2‐y2_/d_xy_ orbitals of 2H‐MoS_2_. This destabilizes the 2H‐MoS_2_ structure and prompts the reorganization of the Mo 4d orbitals, causing the transformation of MoS_2_ from the 2H phase to the 1T phase.^[^ ^21^ ^]^ Consequently, the electronic state of Mo 4d is crucial for determining the electrical properties and phase states of MoS_2_. Any approach capable of adjusting the electronic state of Mo 4d orbitals can trigger the phase transition of MoS_2_.The preparation process of CuS@MoS_2_ can be realized by a two‐part approach: (1) CuMo bimetallic oxide nanosheet precursors were obtained by co‐precipitation method in the presence of polyvinylpyrrolidone (PVP) with Cu(NO_3_)_2_·3H_2_O and Na_2_MoO_4_·2H_2_O as metal ion sources. In the solution system, the PVP molecules can not only adsorb on the surface of the crystal core, but also form a protective film on the crystal surface through the spatial site‐blocking effect, preventing the agglomeration between the particles, which makes the CuMo‐precursor have good dispersion. (2) The CuMo‐precursors were vulcanized by hydrothermal reaction in one step, and the precursors molded under surfactant‐free conditions guided the homogeneous growth of bimetallic sulfide heterogeneous nanosheets during the hydrothermal vulcanization process. For comparison, pure MoS_2_ and CuS nanoflowers were also synthesized using similar processes, respectively (Figures S1 and S2, Supporting Information). As shown in Figure S3 (Supporting Information), the scanning electron microscope (SEM) images of the CuMo‐precursors are highly dispersed irregular nanoflakes with a length of ≈1 µm. And the Cu, Mo, and O elements are uniformly distributed in them.

**Figure 2:**
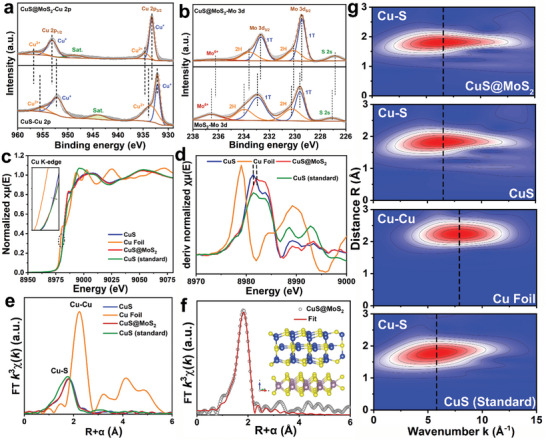
Characterization of fine atom coordination environments. a) High‐resolution XPS spectra of Cu 2p for CuS@MoS_2_ and CuS; b) High‐resolution XPS spectra of Mo 3d for CuS@MoS_2_ and MoS_2_; c) Cu K‐edge XANES spectra, d) first‐derivative XANES curves, e) Fourier transformed (FT) *k* ^3^‐weighted χ(*k*)‐functions at the *R* space, f) EXAFS fitting profiles at the and *R* space (inset showing the optimized structure model). g) wavelet transform (WT) EXAFS plots.

Figure 1b–c shows the SEM images of the CuS@MoS_2_ heterostructure. It can be observed that the homogeneous nanosheets are uniformly distributed in the form of nanoflowers with a particle size of 1 µm without obvious agglomeration. This suggests that CuS@MoS_2_ is gradually grown outward through the CuMo precursor as a nucleation template. Besides, by comparing the morphology with that of pure MoS_2_ nanoflowers, the CuS@MoS_2_ nanoflowers are similar in morphology and show an obvious increase in particle size, which may be attributed to the presence of Cu^2+^ that reduces the activity of the nucleation sites and makes nucleation relatively difficult, leading to an increase in the particle size, and the large nanosheet folds further increase the contact area between the material and the electrolyte.^[^ ^31^ ^]^ Figure S4 (Supporting Information) shows the nitrogen adsorption/desorption isotherms versus pore size distribution curves for CuS@MoS_2_ heterostructure, pure MoS_2_, and CuS. Brunner‐Emmet‐Teller measurements show that the specific surface area of CuS@MoS_2_ hetero‐nanosheets is 19.3 m^2^ g^−1^, which is higher than that of MoS_2_ (11.9 m^2^ g^−1^) and CuS (14.7 m^2^ g^−1^). In addition, the pore volume of CuS@MoS_2_ at large pore distributions (10‐100 nm) is significantly higher than that of MoS_2_ and CuS, which can expose the active sites of the materials to a large extent and accelerate the electrolyte infiltration process, thus improving the Na^+^ storage kinetics. The uniform nanolamellar structure can be further observed from the transmission electron microscopy (TEM) images (Figure 1d–e). The high resolution TEM (HRTEM) image in Figure 1f shows crystal surface with lattice spacing of 0.644 and 0.272 nm clearly, which are attributed to the (002) lattice plane of MoS_2_ and the (006) lattice plane of CuS, respectively (Figure 1g). More importantly, the grain boundaries can be clearly observed, which suggests that heterojunctions were successfully synthesized. Notably, the lattice plane spacing of MoS_2_ is slightly increased compared to the standard 0.62 nm, which tentatively indicates the 1T phase transition of MoS_2_.^[^ ^28^ ^]^ As shown in Figure S5 (Supporting information), the 2H phase displays some hexagonal lattice regions, while the 1T phase presents triangular lattice regions, demonstrating the coexistence of the 1T and 2H phases in the CuS@MoS_2_ heterostructure. Selected area electron diffraction (SAED) further confirms that the sulfide products consist of MoS_2_ and CuS (Figure 1h). The uniform distribution of Cu, Mo, and S elements can be confirmed by elemental mapping from Figures 2i and S6 (Supporting information). In order to further identify the sulfide products, they were analyzed by X‐ray diffraction (XRD) and Raman spectroscopy. As shown in Figure 1j, all the diffraction peaks in the XRD pattern of CuS@MoS_2_ completely coincide with those of MoS_2_ and CuS, respectively, which confirms the coexistence of CuS and MoS_2_ in the heterogeneous structure. In the Raman spectra (Figure S7, Supporting information), for CuS@MoS_2_, in addition to the E_2_ _g_ and A_1_ _g_ peaks at ≈373.0 and 401.1 cm^−1^ representing the vibrational modes of 2H‐MoS_2_, there is a significant increase in the J_1_ (144.3 cm^−1^), J_2_ (236.5 cm^−1^), E_1_ _g_ (279.4 cm^−1^), and J_3_ (336.2 cm^−1^) peaks representing the typical vibrational modes of 1T‐MoS_2_, which further indicates the successful structural transition of MoS_2_ from the 2H phase to the 1T phase.^[^ ^17^, ^19^ ^]^ Figure S8 (Supporting Information) shows the thermogravimetric analysis (TGA) curves of CuS@MoS_2_ composites, pure MoS_2_ and CuS under inert gas (N_2_). The CuS@MoS_2_ has the least amount of heat loss, which indicates the high chemical stability. Electron paramagnetic resonance (EPR) further confirms the electronegativity‐induced vacancies, as shown in Figure 1k. The MoS_2_ and CuS@MoS_2_ observe a signal with a g value of 2.003, which is caused by the unpaired electrons captured by the anion vacancy.^[^ ^32^ ^]^ Among them, the CuS@MoS_2_ heterostructure has the highest resonance signal intensity, indicating that the highest concentration of defects is generated in the crystal structure. This can be attributed to the rational disorder and distortion of the structure at the heterojunction interface caused by the formation of heterojunctions in the two crystal structures.^[^ ^33^ ^]^ The vacancy‐rich sites may provide additional Na^+^ storage sites and modulate the electronic structure and interfacial properties of CuS@MoS_2_, thus lowering the ionic diffusion energy barrier and generating a high pseudocapacitance effect.^[^ ^32^, ^34^, ^35^, ^36^ ^]^ To further analyze the occupancy of the two crystalline phases in the CuS@MoS_2_ heterostructure, its refined XRD is shown in Figure 1l (Table S1, Supporting Information). The mass ratios of MoS_2_ and CuS in the CuS@MoS_2_ heterostructure are about 28.11 wt.% and 71.89 wt.%, respectively.

In order to further analyze the electron flow and atomic chemical states in the heterostructures, XPS spectroscopic and X‐ray absorption fine structure (XAFS) at the Cu K‐edge were performed for CuS@MoS_2_ and other samples. As shown in **Figure** **2a**, the Cu 2p peak of CuS@MoS_2_ is shifted toward higher binding energy compared to CuS. In contrast, the Mo 3d spectra were shifted toward lower binding energies compared to MoS_2_ (Figure 2b). The fine spectra of Mo 3d show a higher 1T phase share in CuS@MoS_2_ compared to MoS_2_, which further indicates that the heterojunction formation has successfully realized the synthesis of 1T‐rich phase MoS_2_. The binding energy shifts of the Cu 2p and Mo 2p peaks indicate the transfer of electrons from CuS to MoS_2_.^[^ ^32^, ^36^, ^37^ ^]^ In addition to this, Figure S9 (Supporting information) shows the high‐resolution XPS spectra of S 2p for the three samples. Figure 2c demonstrates the X‐ray absorption near edge structure (XANES) spectra. Compared to CuS nanosheets, CuS@MoS_2_ nanosheets show a more similar XANES profile, with the absorption edge of CuS@MoS_2_ nanosheets showing a slight shift toward higher binding energy. The position of the absorption edge is related to the average oxidation state of the absorbing atoms, which indicates a slight increase in the Cu valence state in CuS@MoS_2_. The XANES first‐order derivatization curves shown in Figure 2d further indicate that the Cu species in the CuS@MoS_2_ nanosheets have elevated valence states and lost some electrons, which side‐steps the electron transfer from Cu to Mo.^[^ ^38^, ^39^ ^]^ The extended X‐ray absorption fine structure (EXAFS) spectra obtained from the XAS signals were transformed in R‐space and K‐space as shown in Figure 2e with Figure S10 (Supporting information). The CuS@MoS_2_ heterogeneous nanosheets exhibited EXAFS oscillation functions in the low wavelength range like those of pristine CuS nanosheets. Both CuS@MoS_2_ nanosheets and pure CuS show a FT‐EXAFS main peak at 1.78 Å, which is attributed to scatter of the first coordination layer of the Cu‐S.^[^ ^40^ ^]^ The peak value of the main peak of CuS@MoS_2_ nanosheets is slightly lower than that of pure CuS. According to the fitting structural parameters, the coordination number of Cu in CuS@MoS_2_ nanosheets is 3.8, which is slightly lower than 3.9 in CuS, indicating that the number of coordination atoms S in heterostructures is reduced, which is consistent with the result about the high concentration of S vacancy in EPR. From the perspective of field stabilization energy, the lower coordination number may make the central atom form a more stable electronic structure.^[^ ^41^ ^]^ Figures 2f, S11 and S12 (Supporting information) show the results of quantitative EXAFS fitting of the Cu atomic configurations of extracted CuS@MoS_2_ nanosheets to those of pure CuS. The structural parameters are listed in Table S2 (Supporting information). The fitted curves are in good agreement with the experimental spectra, revealing the precise atomic structure. The optimized structural model is shown in the inset of Figure 2f, indicating that the electrons of Cu in CuS@MoS_2_ are transferred and have strong interactions with MoS_2_. Finally, the powerful and informative wavelet transform (WT) is utilized to map the 2D representation of the EXAFS spectral signals in order to discover the high‐resolution features in both k‐space and R‐space. As shown in Figure 2g, due to the scattering of Cu‐S bonds, the WT contour map of CuS@MoS_2_ nanosheets showed a maximum peak value at 5.89 Å^−1^, which showed no change compared with pure CuS, indicating that the length of Cu‐S bonds did not change after the formation of heterojunctions.

### 2.2 DFT Theoretical Calculations

**Figure 3:**
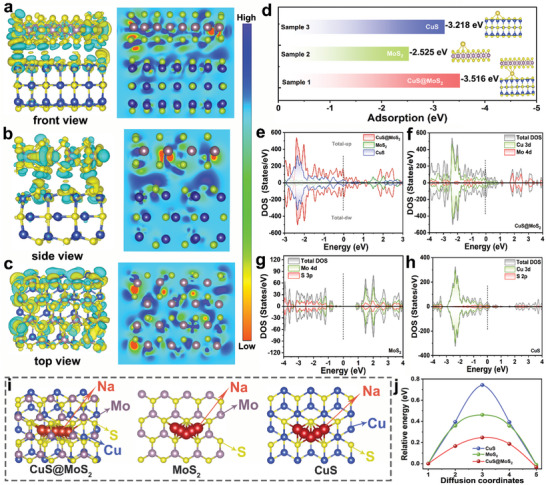
DFT calculations. a) Front, b) side, and b) top views of the calculated charge and density 2D projection of charge density contours difference for CuS@MoS_2_ heterostructures. d) Na^+^ adsorption energy of CuS, MoS_2_, and CuS@MoS_2_. e) DOS of CuS, MoS_2_, and CuS@MoS_2_. The partial density of states (PDOS) of f) CuS, g) MoS_2_ and h) CuS@MoS_2_. i) Schematic diagram of the migration path of Na^+^ in the CuS, MoS_2_, and CuS@MoS_2_ interlayers. j) The diffusion energy barriers of Na^+^ in CuS, MoS_2_, and CuS@MoS_2_, respectively.

DFT calculations are a powerful tool for the prediction of material properties prior to performance testing. In this work, the electron transfer direction in CuS@MoS_2_ heterostructure was verified by DFT calculations and predicted the effect of a strong built‐in electric field on their sodium storage properties (**Figure** **3**). As shown in Figure S13 (Supporting information), the computational model was optimized using CuS with exposed (006) crystal surface and MoS_2_ with (002) crystal surface. Based on the differential charge densities with their 2D color cross sections in Figure 3a–c, it is intuitively clear that a large amount of charge aggregation is generated at the heterointerface with MoS_2_ around in the CuS@MoS_2_ heterostructure, while a localized loss of electrons is manifested on the CuS structure. This result strongly suggests the existence of a built‐in electric field at the CuS@MoS_2_ heterointerface with the direction from CuS to MoS_2_, that CuS can give some of its electrons to Mo, and this interfacial electron transfer should be able to reorganize the Mo 4d orbitals of MoS_2_ to facilitate the formation of its 1T phase. Based on the analysis of Bader charge and charge density differences (Figure 3a–c, Table S3 (Supporting information)), the electron‐rich Cu can provide a fraction of 0.03 e^−^ of electrons to Mo, which leads to a phase transition from 2H to 1T. In addition to this, Figure 3d presents adsorption energy calculations for the optimized Na^+^ adsorption structures of CuS@MoS_2_, MoS_2_, and CuS (Figure S14, Supporting Information). The adsorption energy (ΔEa) of CuS@MoS_2_ for Na^+^ is ‐3.516 eV, which is much smaller than that of MoS_2_ (‐2.525 eV) and CuS (‐3.218 eV). Apparently, these heterostructures based on bimetallic sulfides can significantly enhance the ability to capture Na^+^. In addition, the differences in the density of states of the three configurations were investigated (Figure 3e–h). Figure 3e shows the total density of states (DOS) of CuS@MoS_2_, MoS_2_, and CuS. The band gap widths near the Fermi energy level show the semiconducting nature of MoS_2_ and the conductive properties of CuS. And the DOS intensity of CuS@MoS_2_ near the Fermi energy level is much higher than that of CuS and MoS_2_, which implies that the heterostructures have excellent conductive properties. For CuS@MoS_2_ (Figure 3f), the DOS in the valence band is mainly dominated by Cu 3d, whereas the DOS in the conduction band is mainly dominated by Mo 4d, which suggests that the enhanced Mo 4d exerts a decisive influence on the conductivity of the heterostructure. A localized crossover between Cu 3d and Mo 4d was observed near the Fermi energy level, which indicates an energy level mixing phenomenon in it, suggesting a strong stability of the CuS@MoS_2_ heterostructure. In addition to that, Mo 4d was successfully activated through the partial hybridization of the orbitals between Cu 3d and Mo 4d. What's more, it can be found that the DOS intensity of Mo 4d orbitals is much higher compared with that of MoS_2_ after the formation of heterostructure, which indicates that the Mo 4d orbitals are successfully filled after the introduction of CuS into MoS_2_ to form the heterostructure, which is in line with our expectation. In order to further investigate the kinetic process of Na^+^ diffusion, based on the diffusion paths of Na^+^ along the surfaces of CuS@MoS_2_, MoS_2_, and CuS (Figures 3i and S15 (Supporting information)), the corresponding diffusion energy barriers of Na^+^ were also calculated, as shown in Figure 3j. Obviously, the diffusion energy barriers of CuS@MoS_2_ (0.24 eV) are lower compared with those of the pristine MoS_2_ (0.46 eV) and CuS (0.74 eV), which suggests that there are excellent diffusion kinetics of Na^+^ in the heterogeneous interfaces of CuS@MoS_2_, and that the formation of the built‐in electric field reduces the obstruction of Na^+^ diffusion to a large extent.

### 2.3 Electrochemical Properties of Materials

**Figure 4:**
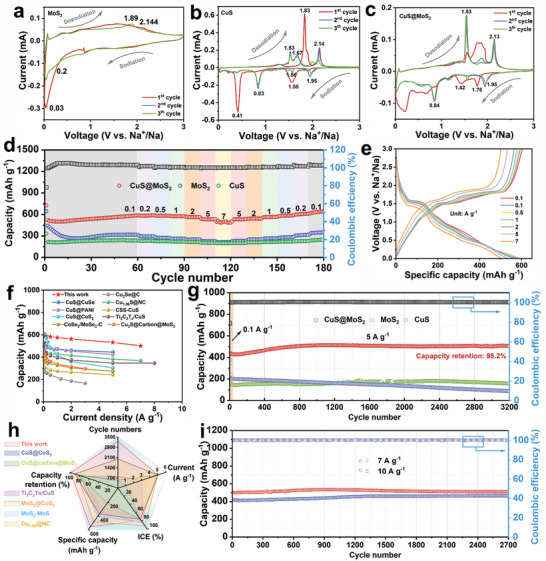
Electrochemical properties in SIB half cell. CV curves of a) MoS_2_, b) CuS, and c) CuS@MoS_2_ at 0.1 mV s^−1^. d) Rate capability of MoS_2_, CuS, and CuS@MoS_2_ at various current densities. e) GCD curves of CuS@MoS_2_ at different current density. f) Rate performance comparison of this work and reported materials for SIBs. g) Long‐term cycling performance of MoS_2_, CuS, and CuS@MoS_2_ at a current density of 5.0 A g^−1^. h) Long‐term cycling performance comparison of this work and reported materials for SIBs. i) Long‐term cycling performance of CuS@MoS_2_ at a current density of 7 A g^−1^ and 10 A g^−1^.

Thanks to the excellent structural features of CuS@MoS_2_ and the guidance of DFT theoretical calculations, it was assembled into a half‐cell with the counter electrode Na foil to evaluate its Na^+^ storage performance. Figure **4a–c** show the cyclic voltammetry (CV) results for the initial three cycles at 0.1 mV s^−1^ for MoS_2_, CuS, and CuS@MoS_2_ electrodes. The CV curves of the MoS_2_ electrode at 0.1 mV s^−1^ are shown in Figure 4a. The redox peak corresponding to MoS_2_ is not obvious, which indicates the poor conductivity of MoS_2_ and the weak response current.^[^ ^42^ ^]^ During the first discharge, a major cathodic peak was observed to be located near 0.03 V, which can be attributed to the conversion reaction of Na_x_MoS_2_ accompanied by the generation of Mo and Na_2_S with the irreversible formation of the solid electrolyte interface (SEI) layer. Starting from the second cycle, the anodic peaks at 2.89 versus 2.144 V during charging can be attributed to the conversion of Mo and Na_2_S to Na_x_MoS_2_ and the regeneration of MoS_2_.^[^ ^27^, ^42^, ^43^ ^]^ Figure 4b shows the CV curves of the CuS electrode at 0.1 mV s^−1^. The cathodic peaks at 1.95 V and 0.83 V are related to the formation of Na_x_Cu_y_S_z_ intermediates. The irreversible strong peak at 0.41 V can be attributed to the formation of the SEI layer and the conversion of Na_x_Cu_y_S_z_ to Cu and Na_2_S. With respect to the anodic scanning process, the peaks at 1.53, 1.67, and 2.14 V in the first cycle are related to the multiple conversion reactions in the process of desodiation.^[^ ^44^, ^45^ ^]^ As shown in Figure 4c, these characteristic redox peaks in Figure 4a,b can be found in the CV curves of CuS@MoS_2_, suggesting that the heterostructure is a biphasic composite composed of CuS and MoS_2_ rather than a single component. The high overlap of the redox peaks in the subsequent cycles indicates that the CuS@MoS_2_ heterostructure is highly reversible in the sodium storage process after activation.

Figure 4d shows the short cycle performance versus rate performance of MoS_2_, CuS, and CuS@MoS_2_. The initial discharge capacity of CuS@MoS_2_ was 725 mAh g^−1^ with 78% ICE. Notably, the capacity of CuS@MoS_2_ increased continuously over 60 cycles, with the capacity rising from 522.2 mAh g^−1^ to 587.37 mAh g^−1^ after 60 cycles. In contrast, MoS_2_ and CuS had no significant activation during cycling and had a much lower discharge specific capacity than that of CuS@MoS_2_ during the whole process. After 60 activation cycles at 0.1 A g^−1^, the reversible capacities of CuS@MoS_2_ are 587.37, 587.3, 582.54, 575.08, 562.24, 532.19, and 500.96 mAh g^−1^ at current densities of 0.1, 0.2, 0.5, 1, 2, 5, and 7 A g^−1^, respectively. When the current density is restored to 0.1 A g^−1^, the electrode maintains a higher reversible specific capacity than the initial cycle, indicating that CuS@MoS_2_ has excellent reversibility. Conversely, the reversible capacity of MoS_2_ and CuS is much lower than that of CuS@MoS_2_ at all current densities. In addition, the galvanostatic charge‐discharge (GCD) curves of CuS@MoS_2_ at different current densities in Figure 4e further demonstrate the excellent rate performance (Figure S16, Supporting information), which is significantly better than that of most previously reported copper chalcogenides and heterostructures for sodium storage (Figure 4f).^[^ ^37^, ^44^, ^46^, ^47^, ^48^, ^49^, ^50^, ^51^, ^52^ ^]^ Thanks to the adsorption capacity of sodium polysulfide provided by the strong internal electric field in the CuS@MoS_2_ heterostructure and the excellent conductivity provided by the 1T‐rich phase MoS_2_ in the structure, the CuS@MoS_2_ electrode can be stably cycling 3200 times at 5 A g^−1^, and can still maintain a high specific discharge capacity of 506.03 mAh g^−1^ after cycling with capacity retention rate of 98.2% (Figure 4g). In contrast, the MoS_2_ and CuS electrodes exhibited a discharge specific capacity of only ≈200 mAh g^−1^ over 3200 cycles, showing poor long‐cycle performance. In addition, we also investigated the cyclic stability of CuS@MoS_2_ heterogeneous anodes at higher current densities (Figure 3i). The reversible capacity of CuS@MoS_2_ is 509.86 mAh g^−1^ / 466.73 mAh g^−1^ after 2700 cycles at 7 A g^−1^ and 10 A g^−1^, respectively. In general, CuS@MoS_2_ has better cyclic stability than most previously reported sodium‐storage anodes based on copper‐based transition metal chalcogenides and heterojunction types (Figure 4h and Table S4, supporting information). To further demonstrate the sodium storage capacity of CuS@MoS_2_, we tested its discharge capacity at different loadings (Figure S17, Supporting information). One can see that it still has an initial discharge capacity of 503.8 mAh g^−1^ even at a high loading of 5 mg cm^−2^, and maintains ≈284 mAh g^−1^ in subsequent cycles, which indicates that CuS@MoS_2_ still has excellent sodium storage capacity at high loading. In order to prove the rigor of promoting Na^+^ storage by CuS@MoS_2_ heterostructures, CuS/MoS_2_ without heterointerface was prepared by grinding pure CuS and MoS_2_ at a mass ratio of 7:3, as shown in Figures S18–S19 (Supporting information). It was found that the reversible capacity of CuS/MoS_2_ without heterogeneous interface is much lower than CuS@MoS_2_ under various current densities. In addition, CuS/MoS_2_ can only cycle 334 times at 5 A g^−1^ and exhibits a low discharge capacity of only ≈200 mAh g^−1^ (Figure S20, Supporting information), which demonstrates the successful construction of the CuS@MoS_2_ heterogeneous interface and the successful regulation of sodium storage behavior by the built‐in electric field.

### 2.4 Sodium Storage Kinetics of CuS@MoS_2_ Heterostructure

**Figure 5:**
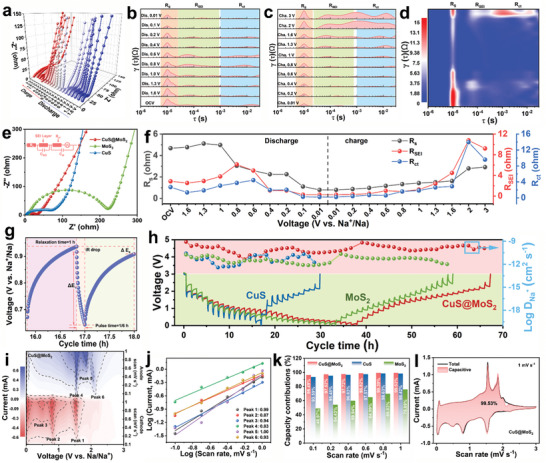
Kinetics analysis of the CuS@MoS_2_ electrode: a) In‐situ Nyquist plots of CuS@MoS_2_ during the initial cycle. b‐c) DRT plots as calculated from the measured impedance data for CuS@MoS_2_ during the first cycle. d) The contour plots of DRT for CuS@MoS_2_ during the first cycle. e) Nyquist plots of MoS_2_, CuS. and CuS@MoS_2_ (the inset show the corresponding electric equivalent circuit). f) The dependencies of R_s_, R_SEI_ and R_ct_ on discharge/charge potential. g) GITT test conditions of CuS@MoS_2_ electrode. h) GITT profiles and corresponding log D_Na_^+^ values of MoS_2_, CuS, and CuS@MoS_2_. i) Contour plots of CV curves for CuS@MoS_2_ at different scan rates. j) Log (i) versus log (v) plots of CuS@MoS_2_. k) Pseudocapacitive contribution for MoS_2_, CuS, and CuS@MoS_2_ at different scan rates. l) Detail capacitive contribution of CuS@MoS_2_ at 1.0 mV s^−1^.

In order to further dissect the reasons for the excellent rate performance and long‐period stability of the CuS@MoS_2_ heterostructure, we have investigated the chemical kinetics of sodium storage by in‐situ/in‐situ electrochemical impedance spectroscopy (EIS) analysis, electrostatic intermittent titration technique (GITT) test, and CV test at different scan rates of 0.1‐1.0 mV s^−1^. As shown in **Figure** **5a**, in‐situ EIS measurements were performed to evaluate the change in conductivity of the CuS@MoS_2_ heterostructured electrodes with cycling. As shown in Figure 5b–d, distribution of relaxation times (DRT) plots were obtained by fitting the in‐situ impedance data from the CuS@MoS_2_ electrodes. As can be seen from Figure 5d, the R_SEI_ and R_ct_ of the CuS@MoS_2_ heterostructure are much smaller than those of MoS_2_ and CuS during cycling (Figures S21–S22, Supporting Information). Figure 5e shows the EIS spectra of MoS_2_, CuS, and CuS@MoS_2_ in the initial state, and the R_ct_ of CuS@MoS_2_ is much smaller than that of MoS_2_ and CuS. As shown in Figure 5f, it can be seen in the variation of the resistance value obtained based on the integration of the DRT map that, throughout the discharge‐charge process, the R_s_, R_SEI_, and R_ct_ of CuS@MoS_2_ are kept at a very low level. Moreover, it can be found that Rs, R_SEI_, R_ct_ of CuS@MoS_2_ all decrease rapidly during the discharge process, and R_ct_ (0.2 Ω) and R_SEI_ (0.27 Ω) reach the lowest value when discharged to 0.01 V. This can be attributed to the narrowing of the material's bandgap by the pre‐insertion of Na^+^ and the formation of transition metals Cu, Mo, etc. which promotes the electron transfer.^[^ ^14^, ^37^ ^]^ The R_s_, R_SEI_ and R_ct_ of CuS@MoS_2_ remain at low levels during charging to 1.6 V, which reflects the very rapid kinetic process of CuS@MoS_2_. Notably, a large increase in R_SEI_ and R_ct_ at the end‐state of charging is observed, which can be attributed to the formation of SEI layer with reversible Na^+^ detachment.

Meanwhile, the diffusion coefficients (D_Na+_) of Na^+^ in MoS_2_, CuS and CuS@MoS_2_ were obtained by galvanostatic intermittent titration technique (GITT) curve fitting (Figure 5g–h). As shown in Figure 5h, CuS@MoS_2_ shows a higher D_Na+_ order of magnitude and charging/discharging time than MoS_2_ and CuS during cycling, which further demonstrates the positive modulation of Na^+^ rapid diffusion by the strong built‐in electric field in the heterostructures of CuS@MoS_2_ with the 1T‐rich phase of MoS_2_, which is compatible with the excellent rate performance. Figure 5i shows the contour cloud map corresponding to CV curves of CuS@MoS_2_ heterostructure at different scan rates of 0.1‐1 mV s^−1^. According to previous studies, the fitting *b* value of the log (*i*) and log (*v*) plots of MoS_2_, CuS, and CuS@MoS_2_ can be used to determine the type of mechanism (battery diffusion control behavior or capacitor surface control behavior) for each redox peak storage. The calculated *b* values of each redox peak are 0.99, 0.87, 0.94, 0.83, 1, and 0.93, respectively (Figure 5j), and the *b* values at all peaks are close to 1, indicating that charge storage is mainly based on pseudo capacitance behavior. In addition, the pseudo‐capacitance contribution of MoS_2_, CuS, and CuS@MoS_2_ to each scan rate is fitted, as shown in Figure 5k. The contribution of CuS@MoS_2_ is greater than 90% at each scan rate and increases with the increase of the scan rate, and is higher than that of MoS_2_ and CuS (Figures S23–S27, Supporting information). It is noteworthy that at the scan rate of 1 mV s^−1^, the pseudo‐capacitance ratio of CuS@MoS_2_ electrode is as high as 99.39% (Figure 5i). The high pseudo‐capacitance indicates that there are a large number of redox active sites in CuS@MoS_2_ and the high utilization rate. This can be attributed to the high S vacancy and strong internal electric field in CuS@MoS_2_, which lead to electron rearrangement at the interface and expose more Na^+^ storage active sites, which enables Na^+^ to enter the electrode material quickly and fully contact with the active site. This is also the main source of CuS@MoS_2_ high discharge capacity and rate performance.^[^ ^53^ ^]^

### 2.5 Exploration of Energy Storage Mechanisms

**Figure 6:**
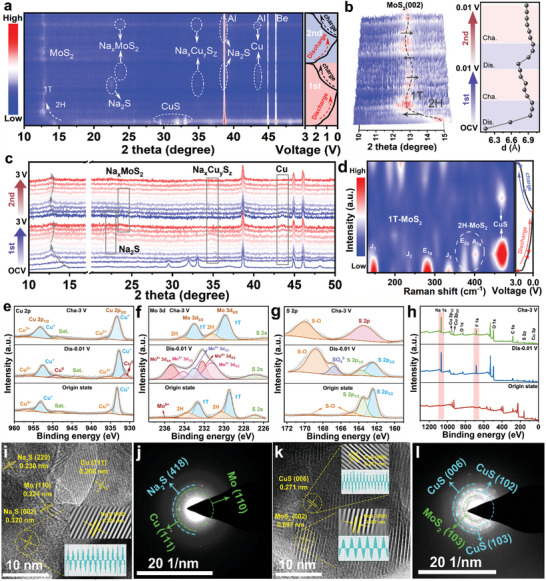
Reaction mechanism exploration: a) Contour plots of in‐situ XRD patterns of CuS@MoS_2_ heterostructure for the first cycle. b) Local peak map of 10–15°. c) Stacked patterns of in‐situ XRD patterns of CuS@MoS_2_ heterostructure for the first cycle. d) Contour plots of ex‐situ Raman spectra of CuS@MoS_2_ heterostructure during the initial discharge/charge cycle. Ex‐situ XPS spectra of e) Cu 2p, f) Mo 3d, g) S 2p, and h) the survey XPS spectrum. HRTEM image and SAED pattern of CuS@MoS_2_ heterostructure at i‐j) fully discharged states and k‐l) charged states in first cycle.

In order to understand the reaction mechanism of CuS@MoS_2_ heterostructures electrode under different discharge and charge states, in‐situ XRD was performed for the first two cycles in a voltage window ranging from 0.01 to 3.0 V. As shown in Figure **6a–c**, the typical diffraction peaks of CuS@MoS_2_ heterostructures can be detected in the in‐situ XRD pattern. During the process from the open circuit voltage discharge to 0.01 V, the peak value of CuS@MoS_2_ gradually weakens and the peak position moves to a lower angle, which corresponds to the Na^+^ inserted into the heterostructures. During the whole cycle, the diffraction peaks of CuS@MoS_2_ completely disappear, and the diffraction peaks of Na_2_S, Na_x_MoS_2_, Na_x_Cu_y_S_z_, and Cu appear, which indicates that a complex intercalation‐conversion process occurs during the sodium insertion process of CuS@MoS_2_. The diffraction peaks of Mo may not be captured because of their low crystallinity and low peaks. In the second cycle, the diffraction peak intensity of each phase is reduced, which may be attributed to the decrease in crystallinity of the electrode material during the cycle. It is worth noting that the diffraction peak of MoS_2_ (002) crystal surface corresponding to 15° has a huge shift to low angle at the initial stage of discharge, and does not have a large reversible shift to high angle in the subsequent process. This can be attributed to the irreversible phase transition of 2H‐MoS_2_ to 1T‐MoS_2_, which means that the initial embedding of Na^+^ widens the layer spacing of MoS_2_ and promotes the irreversible transition of its 2H‐MoS_2_ to 1T‐MoS_2_.^[^ ^28^ ^]^ Figure 6b shows a local peak map of 10–15°. It can be found that the (002) crystal face spacing of MoS_2_ rapidly expands from 6.14 to 6.93 Å with the initial insertion of Na^+^. At the same time, the diffraction peak of MoS_2_ (002) crystal surface has a reversible shift from low to high angle during the subsequent secondary charge and discharge cycle, which also reflects the influence of reversible Na^+^ insertion on the layer spacing of MoS_2_. To further verify the reliability of this irreversible phenomenon, ex‐situ Raman was performed, as shown in Figures 6d and S28 (Supporting information). During the initial discharge phase, all Raman peaks rapidly weaken, indicating that the crystallinity and disorder of the lattice increase with the insertion of Na^+^. In addition, the characteristic peak of CuS@MoS_2_ in the final discharge state disappears, which means that a transformation reaction occurs to form an intermediate phase. In the subsequent charging process, the Raman peak corresponding to 1T‐MoS_2_ gradually reversibly recovers, but no recovery phenomenon of 2H‐MoS_2_ Raman peak is observed. This further confirms the irreversibility of the transition from 2H‐MoS_2_ to 1T‐MoS_2_ phase of the CuS@MoS_2_ heterostructure during the charge and discharge cycle.

In addition, ex‐situ XPS was performed to investigate the valence changes of the CuS@MoS_2_ heterostructure in different charging and discharging states (initial state, discharging to 0.01 V, charging to 3 V). In the high‐resolution spectrum of Cu 2p in the starting state (Figure 6e), two peaks appear at 932.7, 934.6, and 952.7, 955 eV for Cu 2p_3/2_ and Cu 2p_1/2_, which correspond to Cu^+^ and Cu^2+^, respectively. After discharging to 0.01 V, the Cu^0^ peak appeared, corresponding to the production of Cu in the conversion reaction. After charging to 3 V, Cu^0^ disappears, verifying the good sodium storage reversibility of CuS@MoS_2_. For the high‐resolution spectra of Mo 3d (Figure 6e), the peaks corresponding to the 2H and 1T phases of MoS_2_ disappeared after discharging to 0.01 V, and peaks such as Mo^4+^, Mo^5+^, and Mo^6+^ appeared, which corresponded to the generation of Na_x_MoS_2_ and others in the conversion reaction. The result indicated that a complex conversion process took place in the sodium‐embedded process of CuS@MoS_2_. Notably, the area of peaks corresponding to 1T increased after charging to 3 V, which agrees with the results of the ex‐situ Raman, suggesting that a partial phase transition from 2H to 1T has successfully occurred in MoS_2_ during cycling. For the high‐resolution spectra of S 2p (Figure 6g), when the electrode was discharged to 0.01 V, whereas the peak of S‐O increased dramatically, SO_4_^2−^ appeared, which could be attributed to the oxidation of Na_2_S in air and the formation of a small amount of Na_2_SO_4_.^[^ ^37^ ^]^ After charging to 3 V, the peak of SO_4_^2−^ disappears, which is attributed to the consumption of Na_2_S during charging. Figure 6h shows the survey XPS spectrum of different charging and discharging states, and compared to the initial state, new peaks of Na 1s and F 1s appeared in the total spectra, which may be attributed to the formation of fluorinated SEI layer and the residual electrolyte, and the decrease of Na 1s at the end of the charging period also indicates the successful detachment of Na^+^. The ex‐situ HRTEM and SAED measurements also revealed the phase transition of CuS@MoS_2_. Figure 6i–j shows the HRTEM and SAED images of CuS@MoS_2_ when discharged to 0.01 V. Several clear crystal planes can be seen, with the lattice spacing being 0.208, 0.224, and 0.230nm/0.320 nm, respectively. It may be related to the metal Cu (111), Mo (110), and Na_2_S (220)/(002), respectively, which is completely consistent with the in‐situ XRD. At this stage, the corresponding characteristic diffraction rings of Cu, Mo, and Na_2_S nanocrystals in SAED images can be further detected. As shown in Figure 6k–l, CuS and MoS_2_ can still be detected in HRTEM image and SAED diffraction ring when charged to 3.0 V, and the hetero‐interface is also recovered, which indicates that CuS@MoS_2_ has good electrochemical reversibility. It is worth noting that the lattice spacing of MoS_2_ (002) in the final charging state CuS@MoS_2_ is 0.697 nm, which is much larger than the initial state. This is perfectly consistent with the results of in‐situ XRD analysis in Figure 6b, which further reflects the rigor of the phase transition phenomenon.

### 2.6 Electrochemical Performance of Full SIB

**Figure 7:**
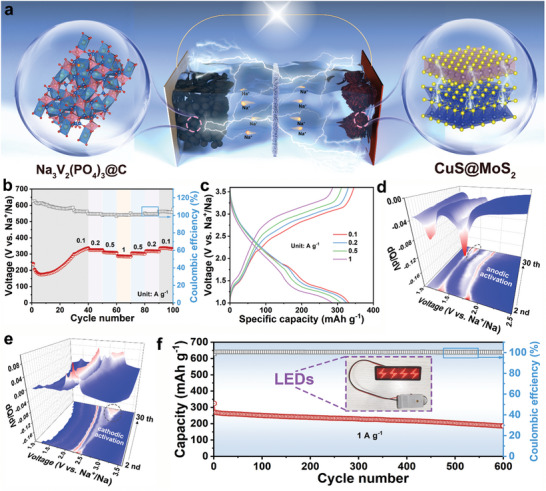
a) Schematic illustration of the CuS@MoS_2_//NVP@C SIB. b) Rate performance of the SIB. c) Charge‐discharge profiles of SIB at different current densities. d‐e) dQ/dV cloud plots of CuS@MoS_2_//NVP@C for the first 30 cycles. f) Long‐term cycling performance of CuS@MoS_2_//NVP@C SIB (Inset: LEDs powered by the SIBs).

Based on the above excellent structural design and half‐cell performance of CuS@MoS_2_, we assembled a CuS@MoS_2_//NVP@C full SIB using commercial Na_3_V_2_(PO_4_)_3_@C (NVP@C) as the cathode and tested its electrochemical performance in the absence of anodic pre‐sodiumation to further evaluate the potential of the application of the CuS@MoS_2_ anode, as shown in **Figure** **7a**. The structural characterization and sodium storage performance of the Na_3_V_2_(PO_4_)_3_@C cathode are shown in Figure S29 (Supporting Information). For optimal full‐cell performance, the operating voltage was chosen to be 1–3.6 V, and the N/P ratio was kept at ≈1:4. Figure 7b shows the rate performance of the CuS@MoS_2_//NVP@C full SIB, with discharge capacities of 331.4, 324.8, 309.7, and 285.8 mAh g^−1^ at current densities of 0.1, 0.2, 0.5, and 1 A g^−1^, respectively. When the current density increases to 10 times of the initial, the capacity of CuS@MoS_2_//NVP@C SIB can still retain 86.2% of the initial. And the capacity did not decay when the current density returned to 0.1 A g^−1^, which indicated the unrivaled multiplicative performance of the CuS@MoS_2_//NVP@C SIB.

The excellent rate performance of CuS@MoS_2_//NVP@C SIB is further demonstrated by the GCD curves at different current densities in Figure 7c, where the plateau position of the GCD curves at different current densities did not change significantly when the SIB activation was completed, indicating the stable sodium storage behavior. It is noteworthy that the specific capacity of the CuS@MoS_2_//NVP@C SIB showed a continuous increase at the beginning of the cycle. In order to analyze the reason for this at a deeper level, the differential capacity curves corresponding to the first 30 cycles of the CuS@MoS_2_//NVP@C SIB were presented in the form of contour plots (Figure 7d–e). During the discharge process, a new peak at 1.5 V appears, which can be attributed to the contribution of Cu collectors to the CuS@MoS_2_ anode.^[^ ^37^, ^54^, ^55^ ^]^ During the discharge process, a large peak gradually appeared at 3 V, which could be attributed to the gradual activation of the anode material NVP@C during the cycles. Therefore, the sustained increase in specific capacity can be attributed to the gradual penetration of electrolyte at both poles and the activation of electrode materials at both poles.^[^ ^12^ ^]^ In addition, the CuS@MoS_2_//NVP@C SIB can achieve a stable cycle of 600 cycles at 1 A g^−1^ and can easily light up an LED sign (Figure 7f). As shown in Figure 7f, the cycling performance of CuS@MoS_2_//NVP@C SIB outperforms many previous transitions metal sulfide‐based full cells (Table S5, Supporting Information). It has been calculated that the average working voltage of CuS@MoS_2_//NVP@C SIB is ≈1.85 V (Figure S30, Supporting information). In addition, CuS@MoS_2_//NVP@C SIB can also obtain an energy density of 300.92 Wh kg^−1^ at a power density of 869.67 W kg^−1^, which is superior to other energy storage devices (Figure S31, Supporting Information), indicating that CuS@MoS_2_//NVP@C SIB has good energy storage application potential.

## 3 Conclusion

In summary, we designed and successfully constructed a homogeneous CuS@MoS_2_ heterogeneous nanosheets to stably induce the 1T‐rich MoS_2_ transition via electronic modulation and built‐in electric field strategies for sodium ion storage with high rate performance and long cycle life. The phase transition mechanism suggests that the equilibrium electron occupation in the Mo 4d orbital is a core idea to realize the effective phase transition of MoS_2_. DFT and XAFS results illustrate that the electron‐rich CuS at the CuS@MoS_2_ hetero‐interface acts as an electron donor and transfers part of the electrons to Mo, which triggers the reorganization of the Mo 4d orbitals and creates a strong built‐in electric field at the interface to successfully induce the transition from 2H to 2T‐MoS_2_. irreversible phase transition of MoS_2_ from 2H to 1T. In‐situ XRD, ex‐situ Raman, and HRTEM results confirm the high reversibility of 1T‐MoS_2_ during the electrochemical process. In addition, DFT calculations show that the CuS@MoS_2_ heterostructure significantly reduces the Na^+^ diffusion energy barrier, improves the electrical conductivity, and accelerates the Na^+^ storage kinetics. Based on its structural advantages, CuS@MoS_2_ heterogeneous nanosheets have a high initial discharge capacity of 725 mAh g^−1^ at 0.1 A g^−1^ with a Coulombic efficiency of 78%, excellent rate performance (466.73 mAh g^−1^ at 10 A g^−1^) and long cycle endurance (506.03 mAh g^−1^ after 3200 cycles at 5 A g^−1^ and 500.03 mAh g^−1^/464.18 mAh g^−1^ after 2700 cycles at 7 A g^−1^/10 A g^−1^). This work provides new ideas and perspectives for the development of high‐performance sodium storage based on 1T‐rich MoS_2_ materials.

## Conflict of Interest

The authors declare no conflict of interest.

## Author Contributions

H.P. and W.M. contributed equally to this work. H.P.: conceptualization, funding acquisition, writing‐review & editing. W.M.: methodology, software, formal analysis, investigation, and writing‐original draft. J.Z.: investigation and methodology. Z. W.: formal analysis. C.Y.: validation. G.M.: resources, funding acquisition, and supervision. Z.L.: resources and funding acquisition.

## Supporting information



Supporting Information

## Data Availability

The data that support the findings of this study are available from the corresponding author upon reasonable request.
