# Supplementary material for: Electronic Modulation and Built‐in Electric Field Strategies in Heterostructures Together Induce 1T‐Rich MoS2 Conversion for Advanced Sodium Storage
Source: Adv Sci (Weinh). 2025 Feb 10;12(13):2417288. doi: 10.1002/advs.202417288 (PMC11967872; doi:10.1002/advs.202417288)
Supplement: Supplementary file 1 — Supporting Information [file ADVS-12-2417288-s001.docx]

Supporting information

**Electronic modulation and built-in electric field strategies in heterostructures together induce 1T-rich MoS_2_ conversion for advanced sodium storage**

*Hui Peng*^‡^*^*^, Wenxing Miao*^‡^*, Jingtian Zeng, Zihao Wang, Chenhui Yan, Guofu Ma^*^, Ziqiang Lei*

Key Laboratory of Eco-functional Polymer Materials of the Ministry of Education, Key Laboratory of Polymer Materials of Gansu Province, College of Chemistry and Chemical Engineering, Northwest Normal University, Lanzhou 730070, China.

^‡^These authors contributed equally to this work.

E-mail: penghui@nwnu.edu.cn (H. Peng); magf@nwnu.edu.cn (G. Ma)

**S1. Experimental section**

***S1.1 Materials***

Copper nitrate trihydrate (Cu(NO_3_)_2_·3H_2_O), Sodium molybdate dihydrate (Na_2_MoO_4_·2H_2_O), thiourea (CH_4_N_2_S), polyvinylpyrrolidone (PVP) and anhydrous ethanol were purchased from Shanghai Aladdin Biochemical Technology Co., Ltd. All chemicals were analytical grade and used without further purification.

***S1.2 Preparation of CuMo precursor***

To prepare the CuMo precursor, 0.3 g of PVP was first dissolved into 300 mL of deionized water to form a homogeneous solution. Then 1.2 g of Cu(NO_3_)_2_·3H_2_O and 1.2 g of Na_2_MoO_4_·2H_2_O were dispersed therein and stirred magnetically for 1 h at room temperature. The light green precipitate was collected by centrifugation and washed with deionized water and ethanol several times. Finally, the precursors were obtained with drying at 60 °C for 6 h.

***S1.3 Preparation of CuS@MoS_2_ Heterostructure***

In a typical synthesis procedure, 0.1 g as-prepared CuMo precursor were dispersed in 20 mL deionized water with magnetic stirring for 0.5 h to form a light green suspension. 0.8 g thiourea was then added into the suspension with magnetic stirring for 10 min to form a yellow suspension. The well-dispersed suspension was then transferred to a 50 mL Teflon-lined stainless-steel autoclave and maintained at 200 °C for 24 h. After cooling to room temperature, the black precipitate was filtered and washed with deionized water and ethanol several times. Finally, the precipitate was obtained by vacuum drying at 60 °C for 6 h. For comparison, the pure CuS and pure MoS_2_ were prepared under the same conditions, using 1.2 g Cu(NO_3_)_2_·3H_2_O and 1.2 g Na_2_MoO_4_·2H_2_O as the precursor, respectively.

***S1.6 Preparation of CuS/MoS_2_***

Based on the refined XRD results of CuS@MoS_2_, the pure CuS and pure MoS_2_ were ground in proportion (7:3) for 10 minutes.

**S2. Material characterization**

The morphology and microstructure of the as-prepared materials were examined by field emission scanning electron microscopy (FE-SEM, Carl Zeiss-Ultra Plus, Germany) and transmission electron microscopy (TEM, FEI Tecnai G2 F20 S-Twin, USA). Raman spectra were collected on a Via Raman spectrometer (Rainie Salt Public Co. Ltd., Britain) with a laser wavelength of 514 nm. The atom coordination environments of the materials were determined by X-ray absorption fine structure (XAFS, Rapid XAFS 2M). The crystallographic structure of the materials was determined by X-ray diffraction (XRD, D/Max-2400, Rigaku) equipped with Cu Kα radiation (k=1.5418 Å). X-ray photoelectron spectroscopy (XPS) measurement was performed on an Escalab 210 system (Germany) with Al Kα radiation source. The Brunauer-Emmett-Teller surface area (BET) of the samples were analyzed by nitrogen adsorption/desorption in a Quantachrome Autosorb IQ3 (USA). The pore size distribution plots were recorded from the desorption branch of the isotherms based on the Barrett-Joyner-Halenda (BJH) model.

During in-situ XRD measurements, an aluminum (Al) foil and a beryllium (Be) foil window were used as the current collectors and the X-ray penetrator, respectively. The data were collected at a scanning rate of 0.8° min^-1^ from 10° to 50° during the first two discharge/charge cyclings. In-situ electrochemical impedance spectroscopy (EIS) measurements were performed on Bio-Logic VSP-300 (French). The data were collected at a current density of 0.05 A g^-1^ during the initial discharge/charge cycling.

**S3. Fabrication of cell devices and electrochemical measurements**

The electrochemical properties of all anode materials were investigated in a CR2032 coin cell. A homogeneous slurry was obtained for the preparation of the working electrode using the active substance, acetylene black and polyvinylidene fluoride (PVDF) mixed homogeneously in the mass ratio of 7:2:1, and a small amount of N-methyl-2-pyrrolidone (NMP) was added and stirred in a small beaker overnight. The slurry was then coated on a clean copper foil using a spatula. After overnight in a vacuum oven at 60 °C, the foil was perforated into discs of 12 mm diameter. The mass loading of the active substance was about 1.1 mg cm^-2^. Finally, in an argon filled glove box (H_2_O < 0.1 ppm, O_2_ < 0.1 ppm), handmade sodium foil was used as the counter electrode, glass fiber (Whatman GF/D) as the separator, and 1.0 M NaPF_6_ in DME as the electrolyte were assembled to form a coin type (CR 2032) half-cell. In the full cell, Na_3_V_2_(PO_4_)_3_@C as cathode material was mixed with acetylene black and polyvinylidene difluoride (PVDF) in a weight ratio of 7:2:1, and the cathode was then obtained by a coating and punching operation on a charcoal-coated aluminum (Al) foil through a procedure like that of CuS@MoS_2_. The mass ratio of the cathode/anode material was about 4. The specific capacity of the full cell in terms of the mass of the anode active materials.

In GITT test, D-value of the two samples were obtained from the formula: [5]

D = $\frac{4}{\Pi\tau}\left( \frac{m_{B}V_{M}}{M_{B}A} \right)^{2}\left( \frac{\Delta E_{S}}{\Delta E_{\tau}} \right)^{2}$ (S1)

where the m_B_ is the mass of the Cu_1.96_S@NC and Cu_1.96_S electrode. M_B_ and V_M_ represent the molar mass, and molar volume of the Cu_1.96_S. A is electrode area and τ represents current pulse duration. ΔE_τ_ is the change of each-step voltage, ΔE_s_ represents steady-state voltage irrespective of IR drop.

On this basis, all galvanostatic charge-discharge (GCD) cycling tests were measured on a NEWARE battery test system (NEWARE, MIHW-200, China) at room temperature with a voltage window of 0.01-3 V. Cyclic voltammetry (CV) tests were performed on a Metrohm Autolab electrochemical workstation (PGSTAT 302).

**S4. Calculation methods**

Density-functional theory (DFT) calculations were performed with the Vienna Ab-initio Simulation Package (VASP) codes 5.4. PAW pseudo-potentials, and the Perdew-Burke-Ernzerhof exchange-correlation functional, and a plane wave cutoff of 400 eV were used in the calculations. All periodic slab calculations were carried out using a vacuum spacing of at least 15 Å. 3×3×1 k-point meshes were sampled for all structures. The convergences of energy and force were set to 10−6 eV and 0.05 eV/Å, respectively. Spin-polarized calculations were used for all the structures.

The calculations were performed using the Vienna Ab-initio Simulation Package (VASP) within the framework of density functional theory (DFT) and the Perdew-Burke-Ernzerhof (PBE) functional. The interactions between ion cores and valence electrons were treated using the projector augmented wave (PAW) potential. A cut-off energy of 400 eV was employed for the expansion of the valence electron wave function, and a convergence force criterion of 0.01 eV Å⁻¹ was used. To account for van der Waals interactions, the DFT-D3 method with Becke-Jonson damping was utilized to address the limitations of DFT in treating dispersion interactions. In order to prevent interactions between periodic layers, a vacuum layer with a thickness of 15 Å was introduced along the z-direction. For the geometry optimizations, a 3×3×1 k-point sampling was selected for structures. Furthermore, the total density of states (TDOS), the partial density of states (PDOS), and the band structures of systems were calculated. To determine the energy barriers for the migration of Na atoms within the layers, the climbing image nudged elastic band (CI-NEB) method was employed. The energy and force convergence criteria used in the structural relaxation were also applied in these calculations.

Regarding the analysis of Na ion adsorption in each system, the adsorption energy (E_ads_) was defined as

E_ads_ = E_Tot_ - E_sub_ - E_Na_

where E_Tot_, E_sub_, and E_Na_ represent the calculated total energies of the substates with the Na atom, the substrates, and the isolated Na atom, respectively.


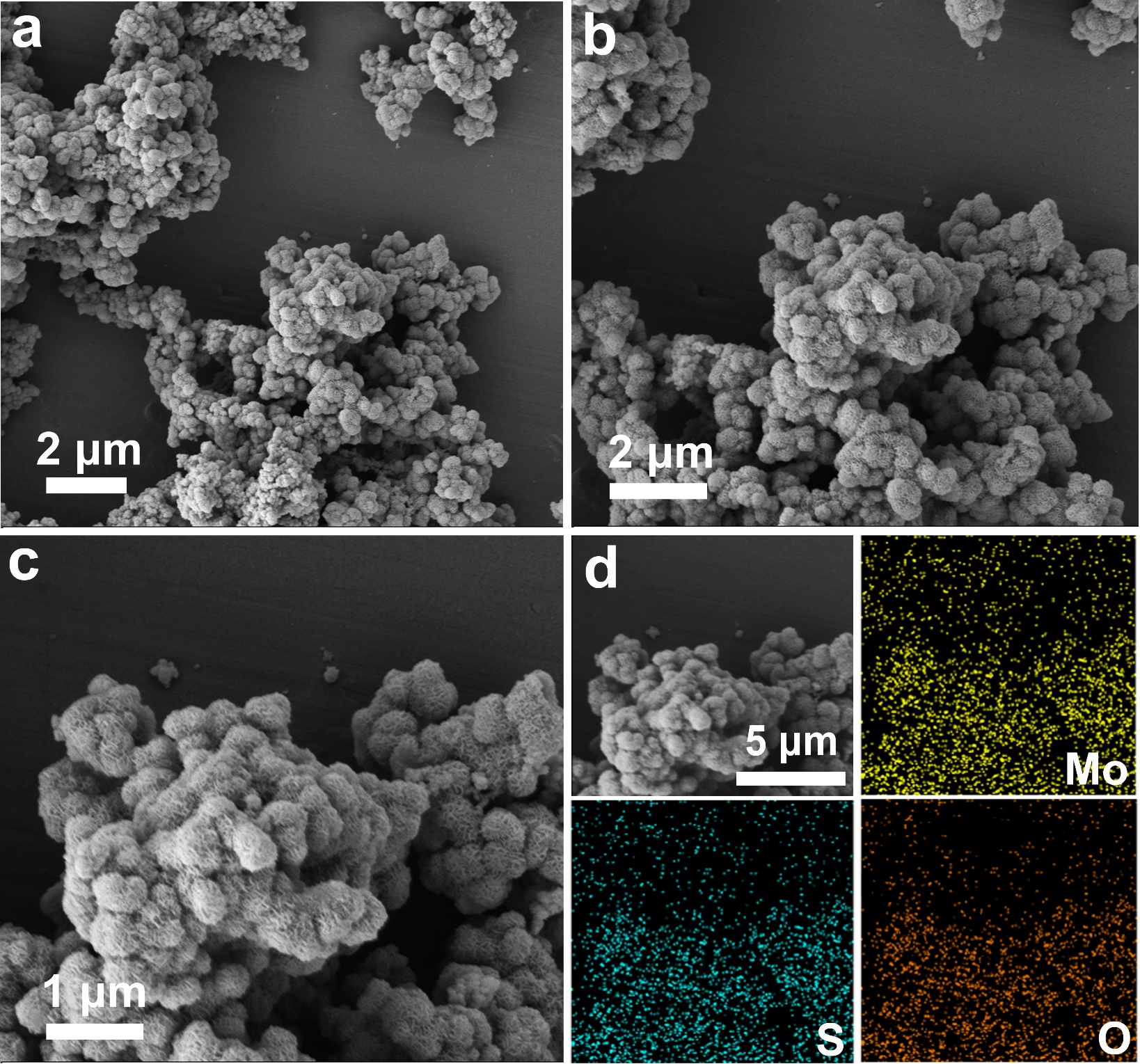


**Figure S1**. (a-c) SEM images and (d) EDS mapping of pure MoS_2_.


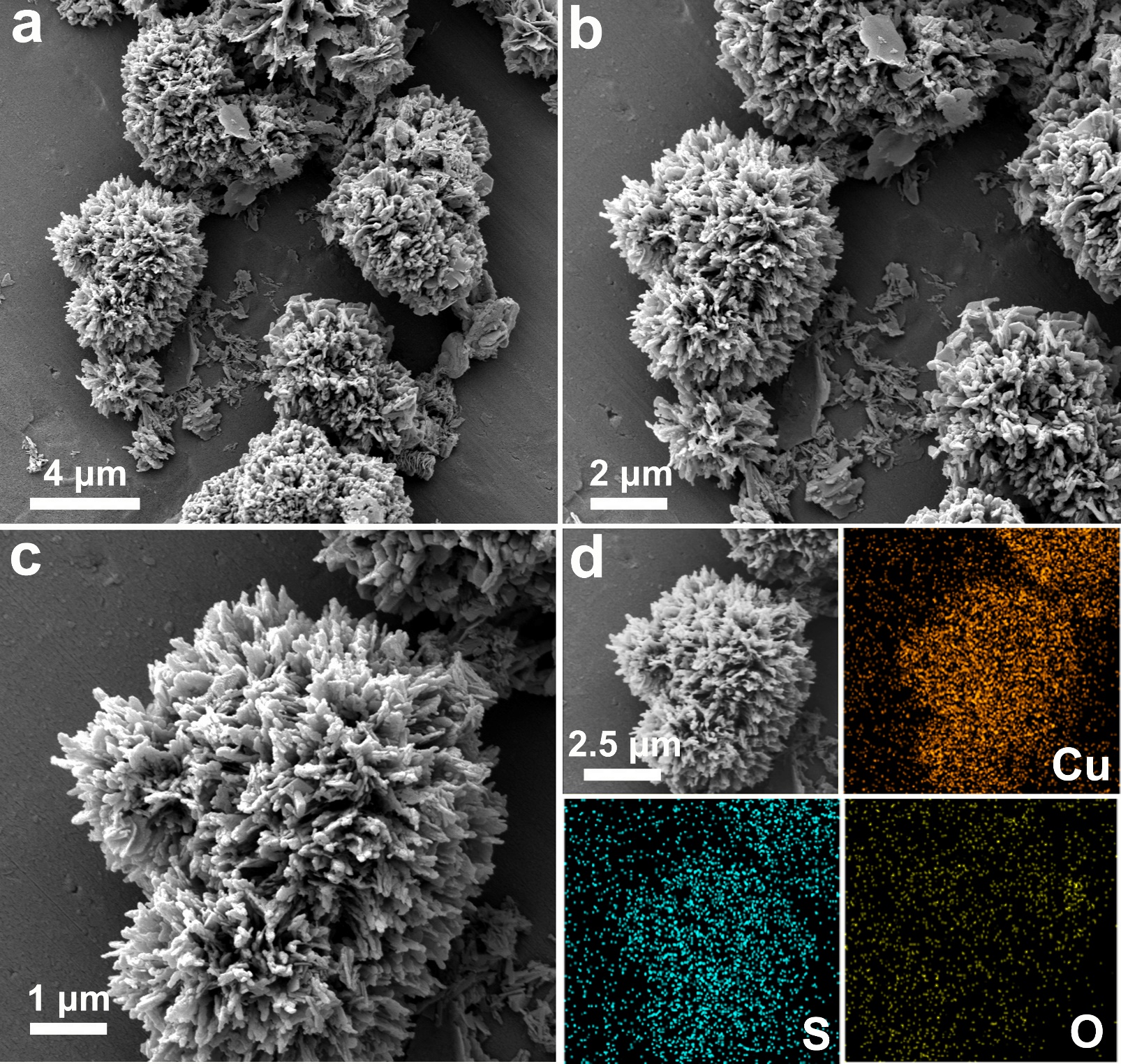


**Figure S2**. (a-c) SEM images and (d) EDS mapping of pure CuS.


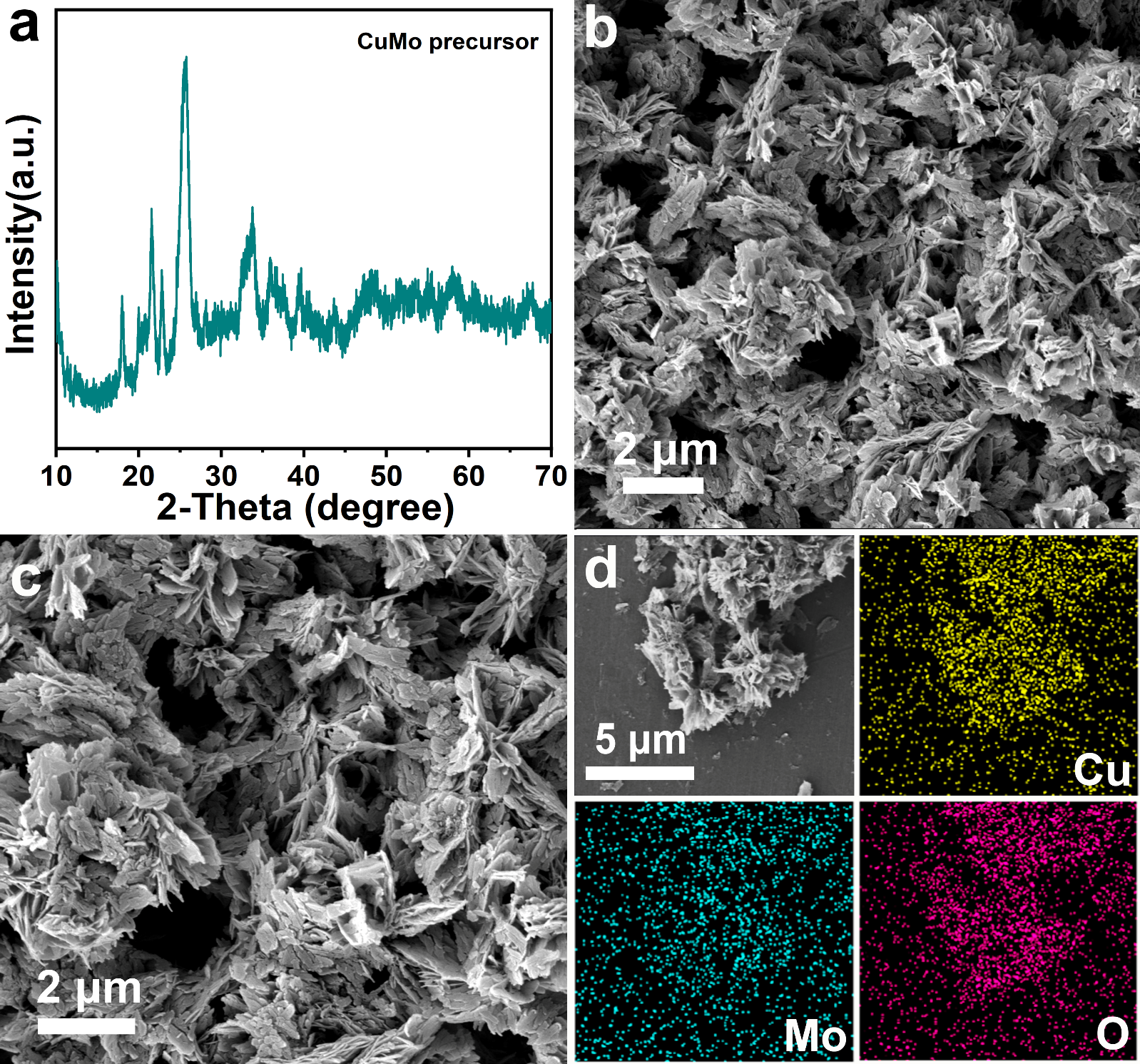


**Figure S3**. (a) XRD pattern, (b-c) SEM images and (d) EDS mapping of CuMo precursor.


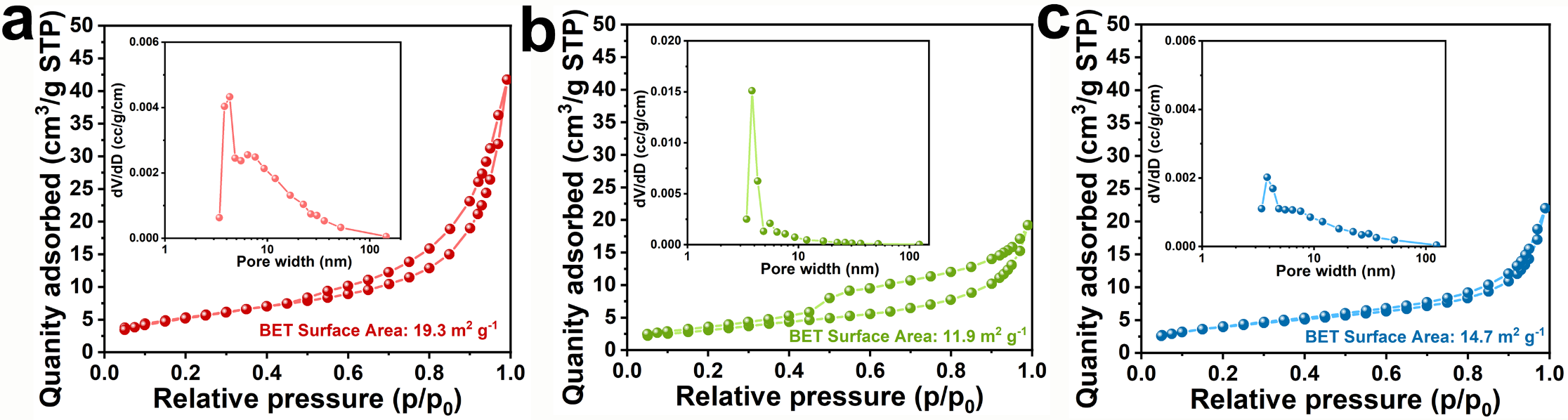


**Figure S4**. Nitrogen adsorption-desorption isothermal curves and corresponding pore size distribution curves of (a) CuS@MoS_2_, (b) MoS_2_ and (c) CuS.


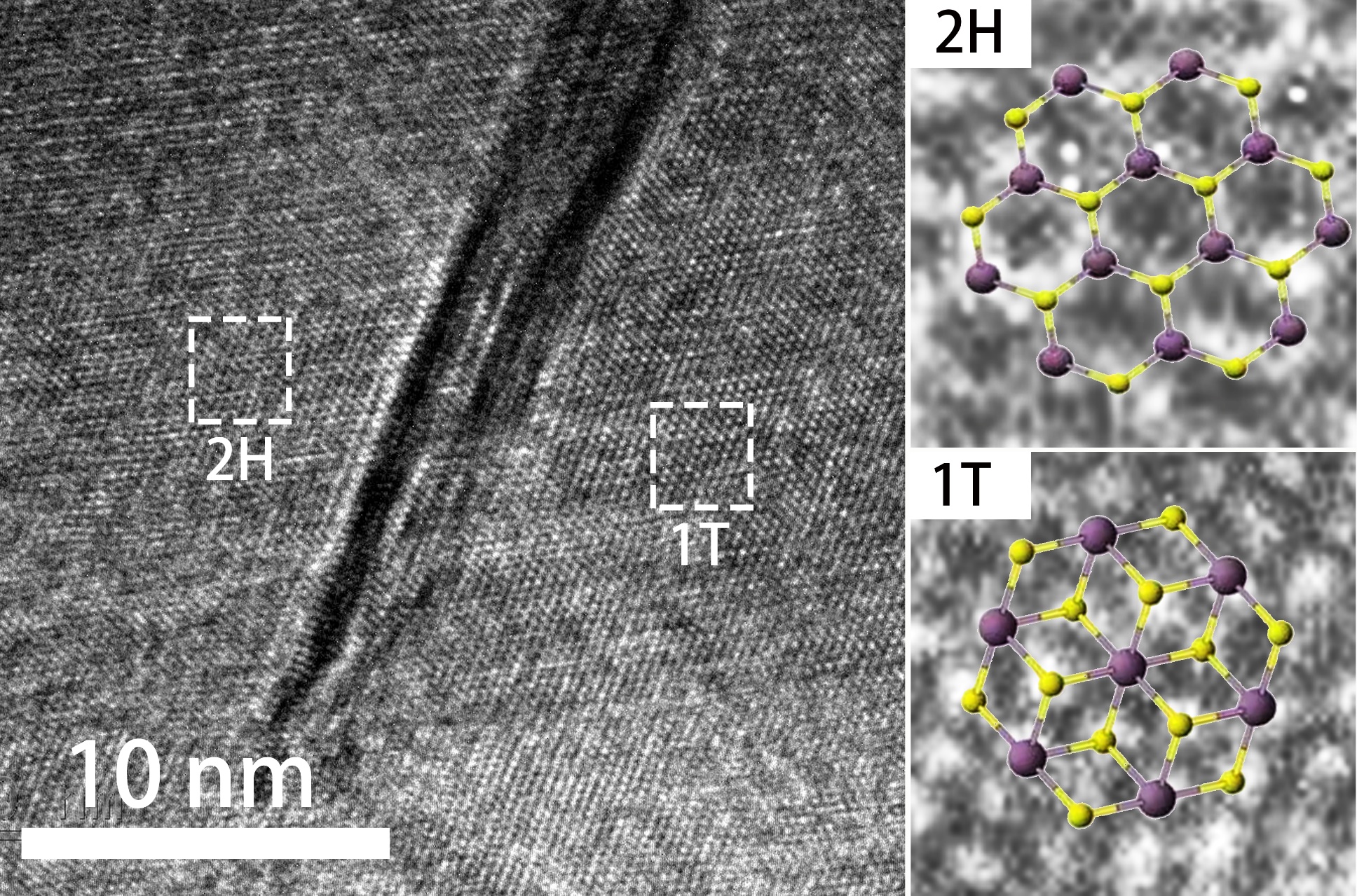


**Figure S5**. The HRTEM of CuS@MoS_2_. The inset images show schematics of 2H-MoS_2_ and 1T-MoS_2_ structures.


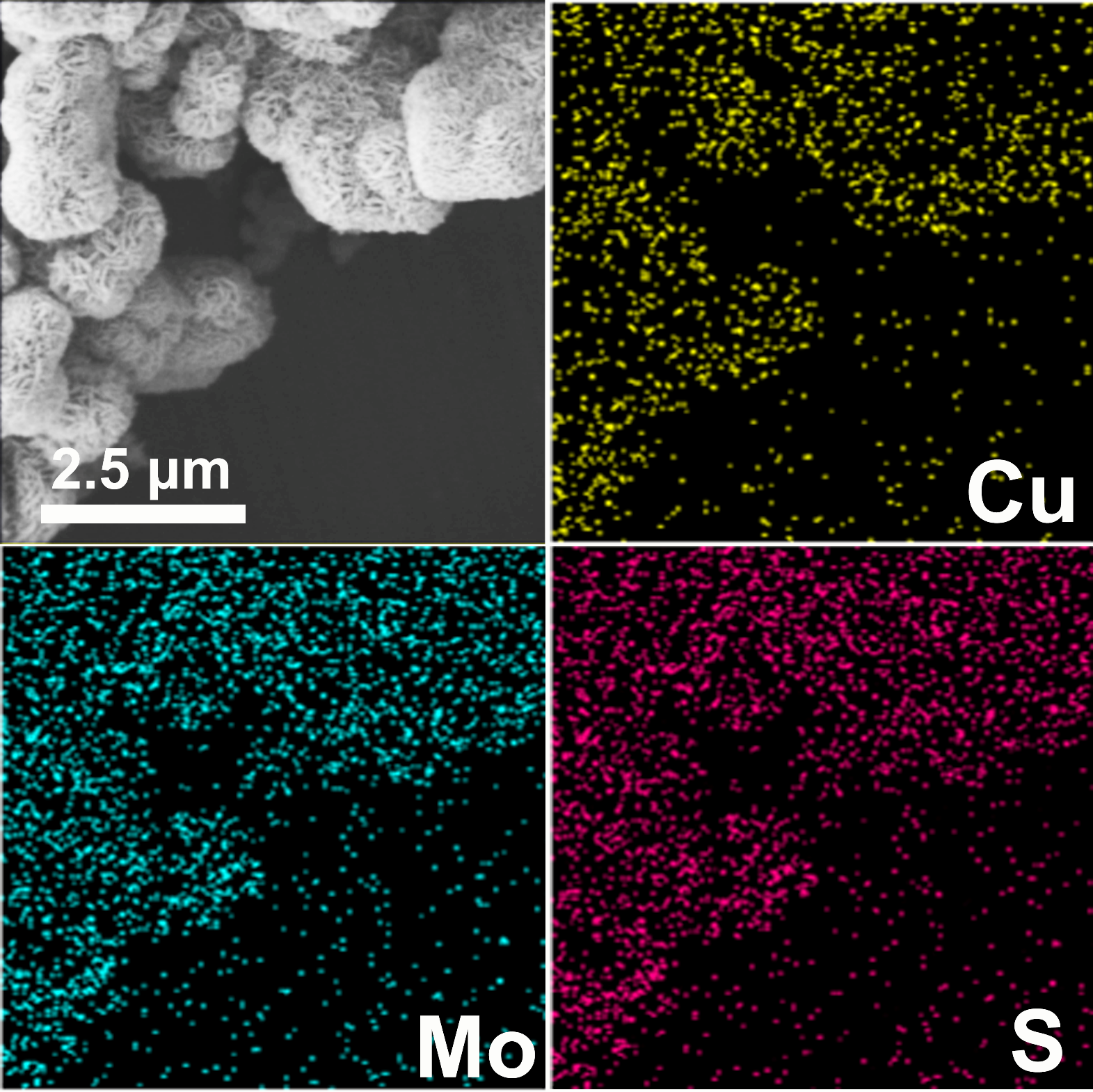


**Figure S6**. EDS mapping of CuS@MoS_2_.


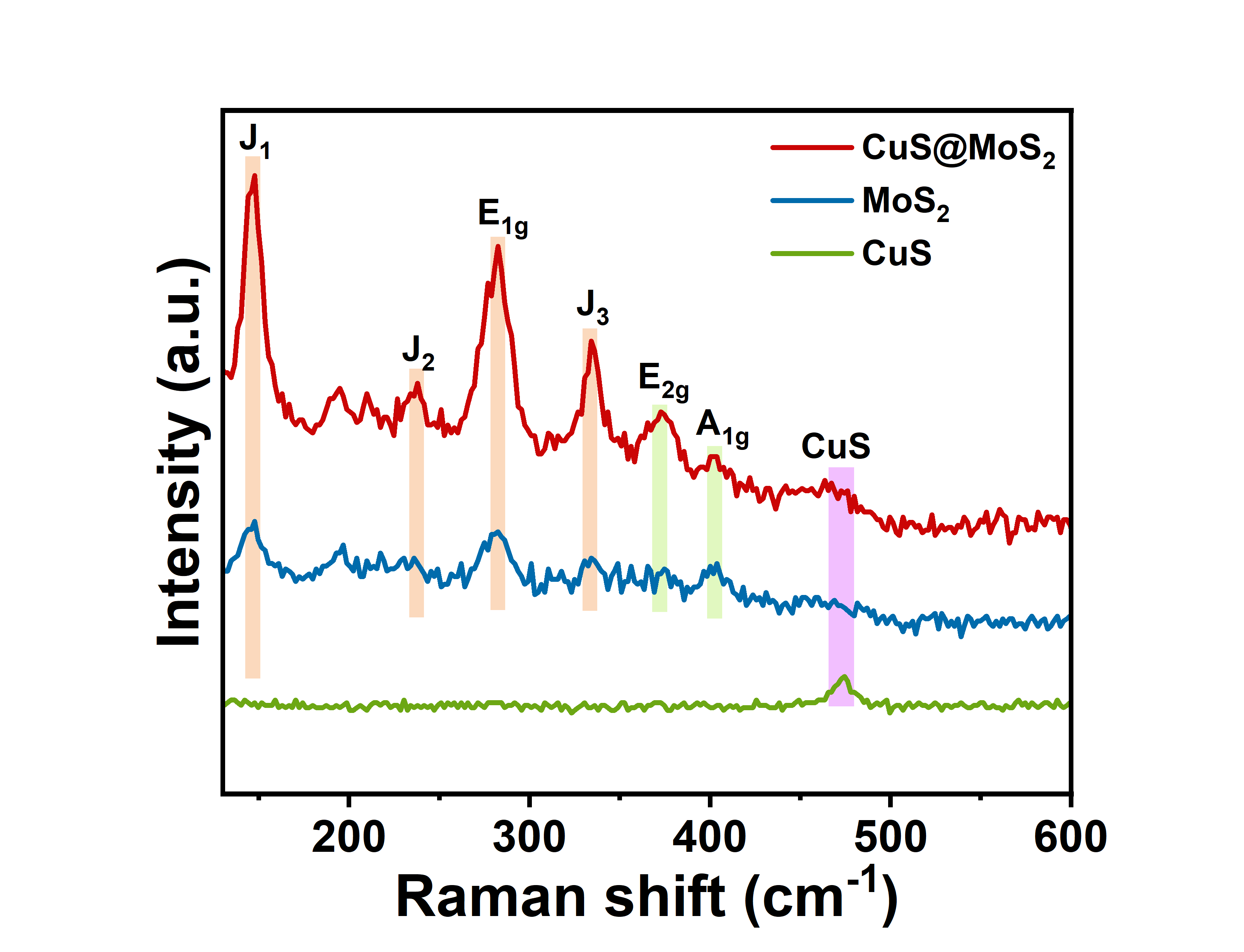


**Figure S7**. Raman spectra of CuS@MoS_2_, MoS_2_ and CuS.


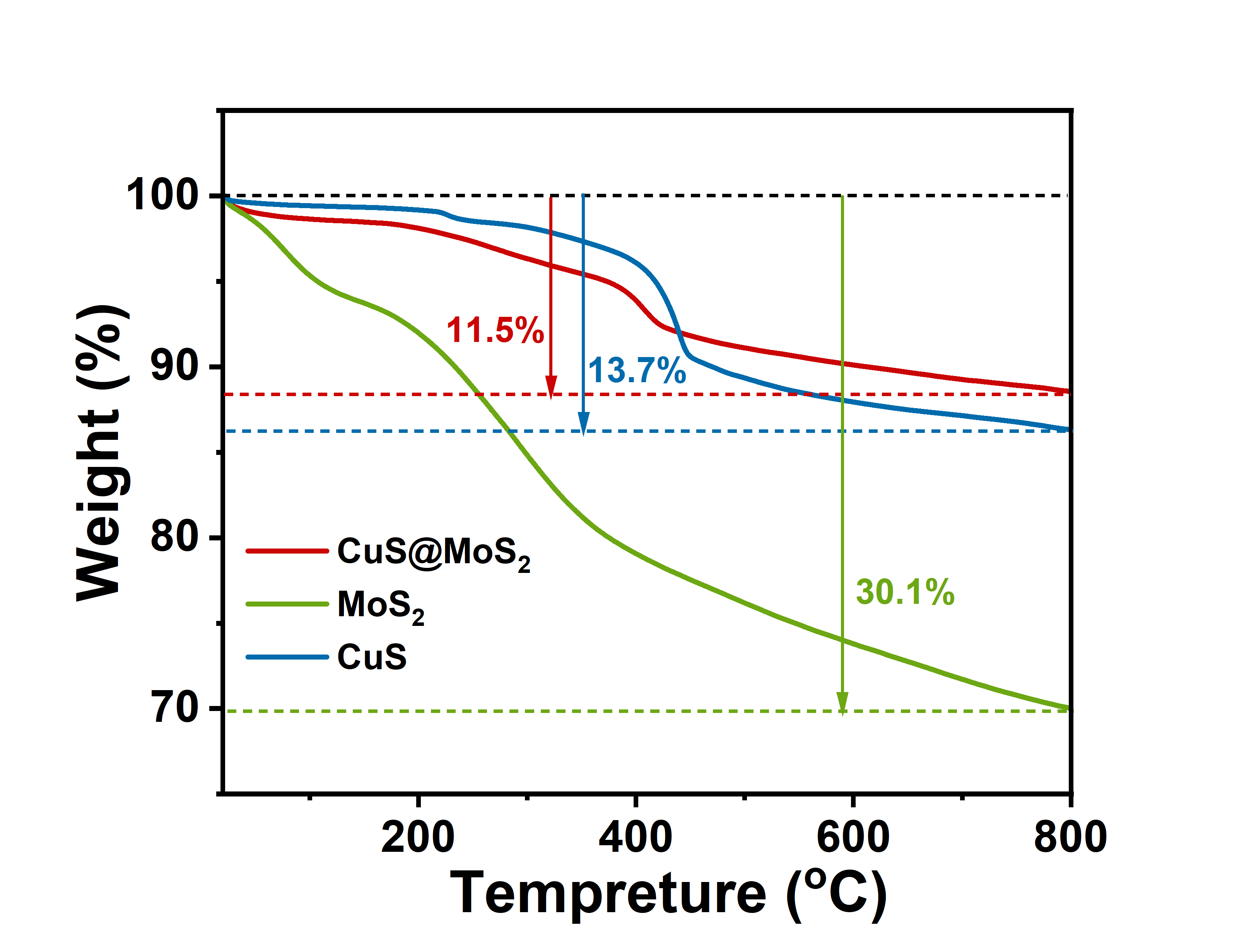


**Figure S8**. TGA curves of CuS@MoS_2_, MoS_2_ and CuS in N_2_ atmosphere.


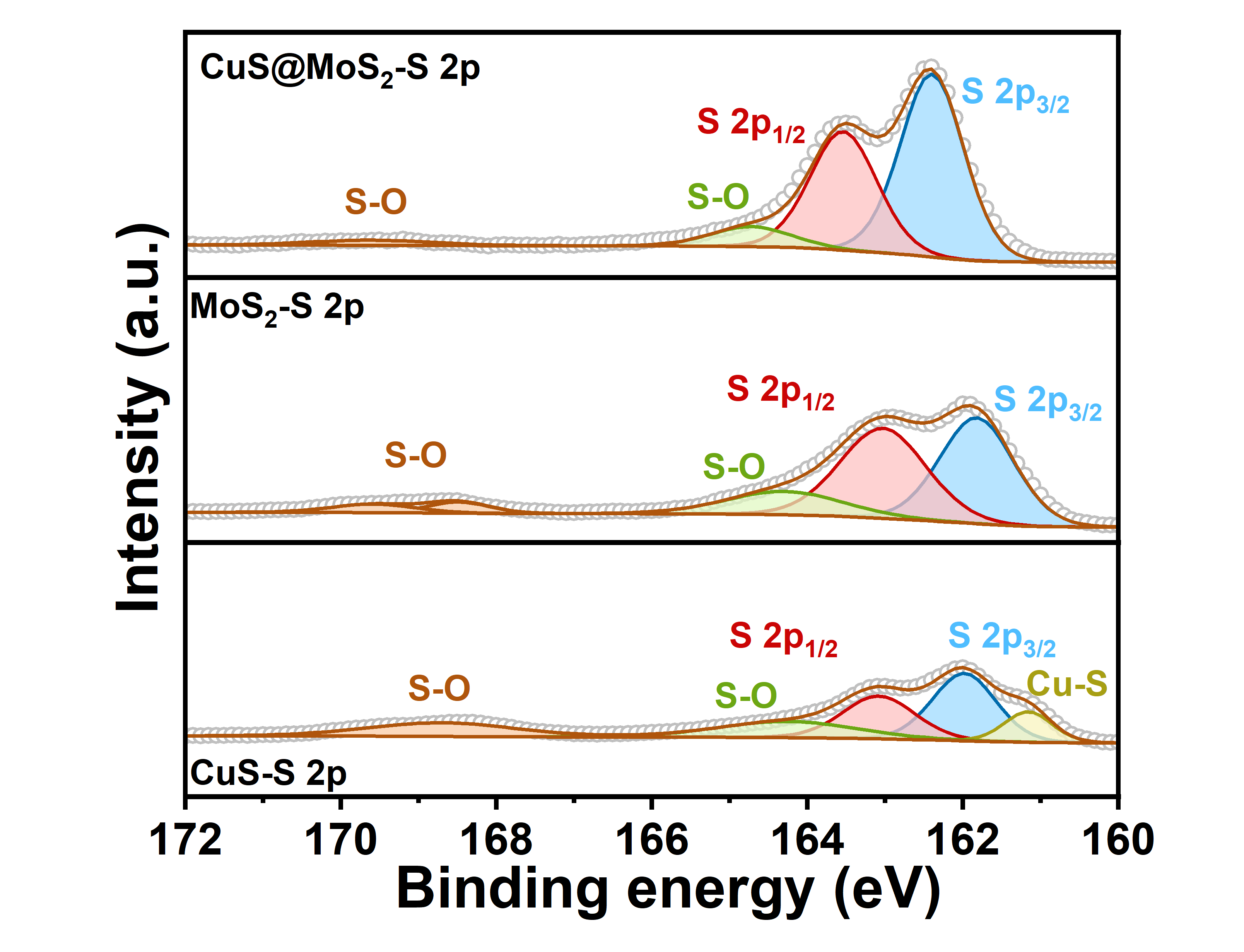


**Figure S9**. High-resolution XPS spectra of S 2p for the CuS@MoS_2_, MoS_2_ and CuS.


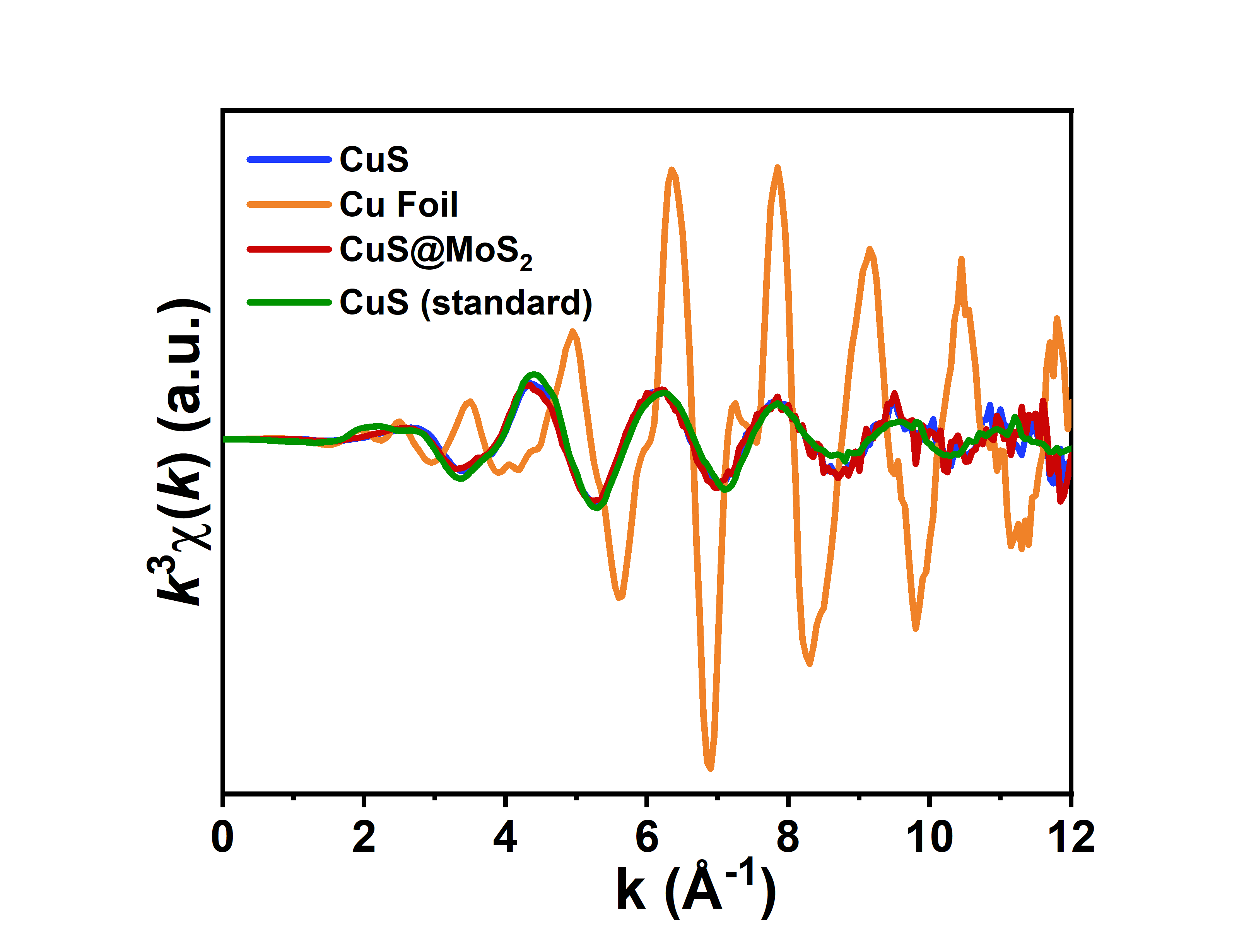


**Figure S10**. Cu K-EXAFS oscillation functions *k*^3^ χ(*k*) at the *k* space.


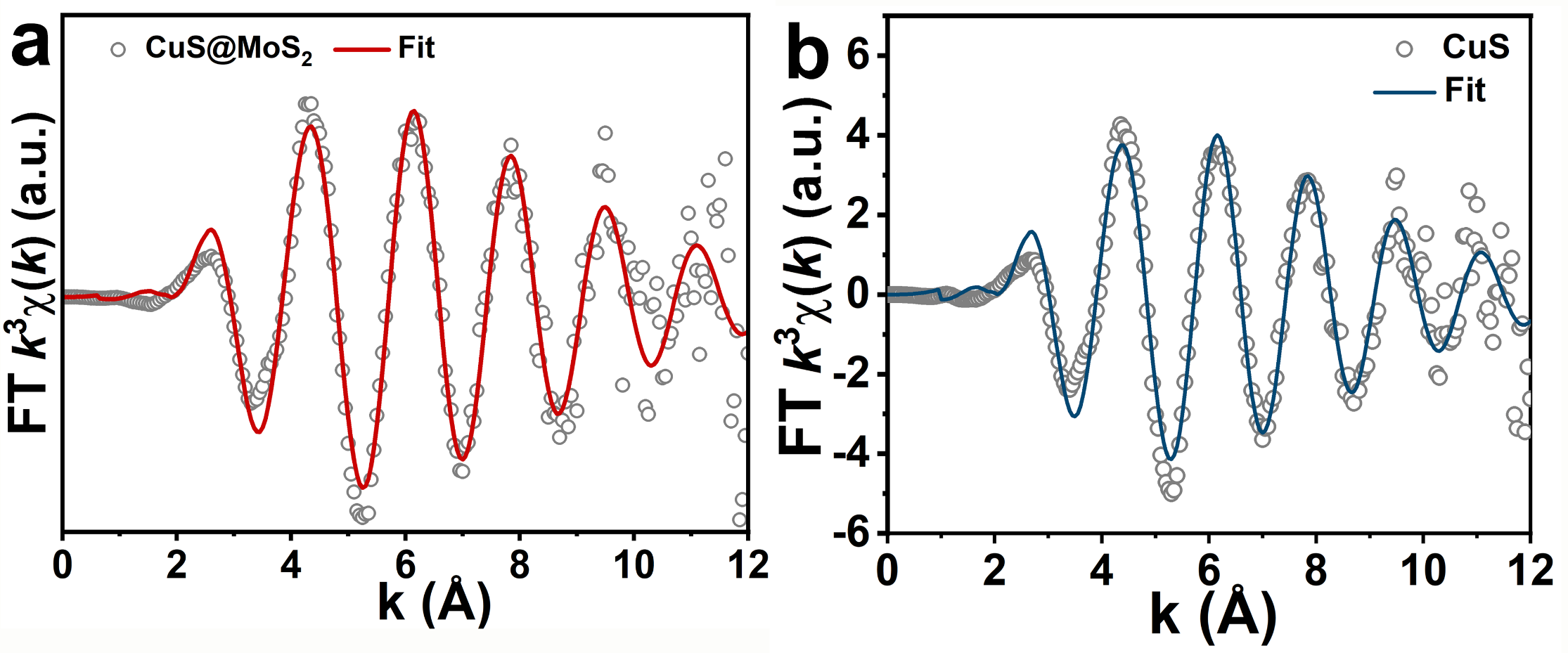


**Figure S11**. EXAFS fitting profiles at the *k* space for the CuS@MoS_2_ and CuS.


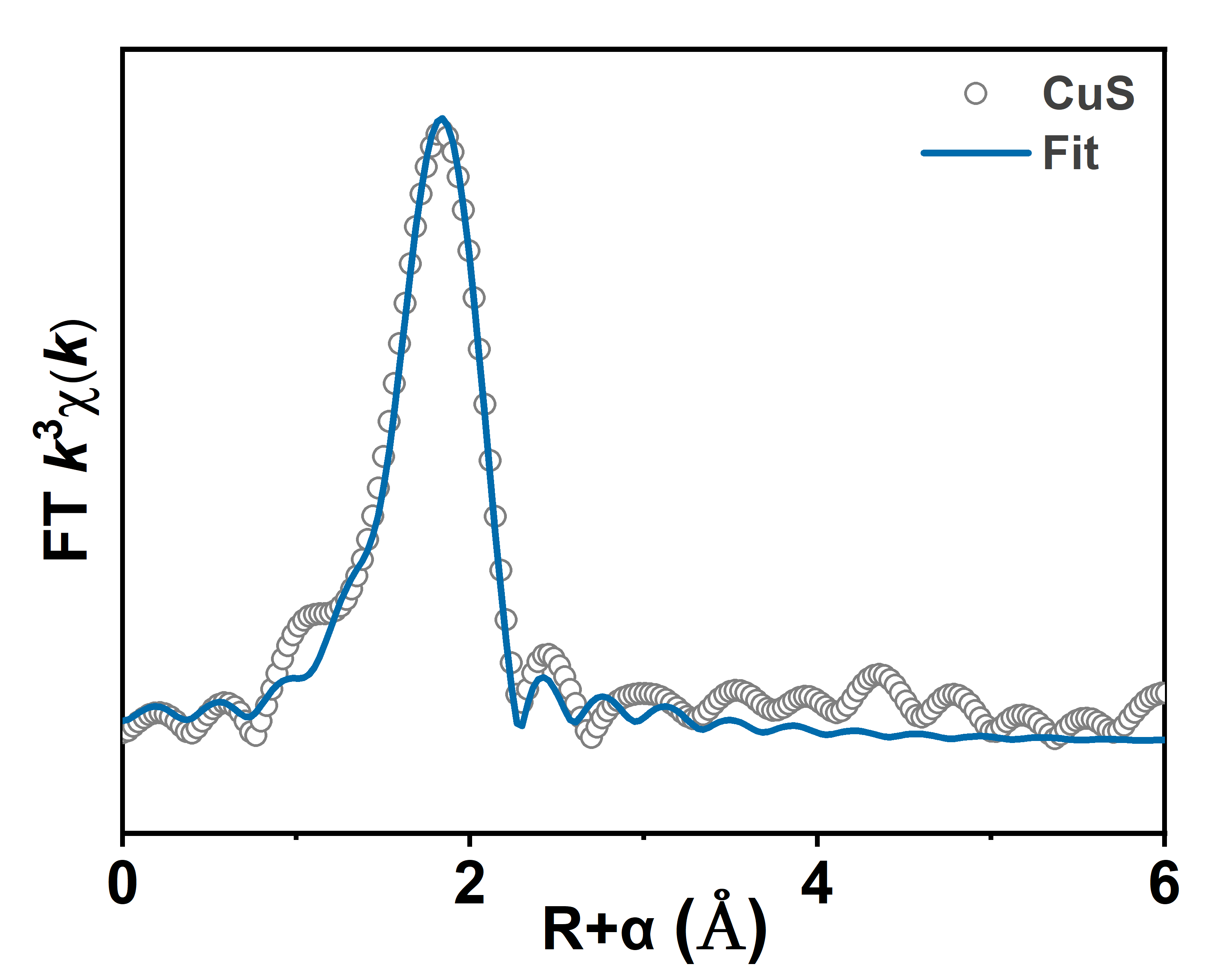


**Figure S12**. EXAFS fitting profiles at the *R* space for the pure CuS.


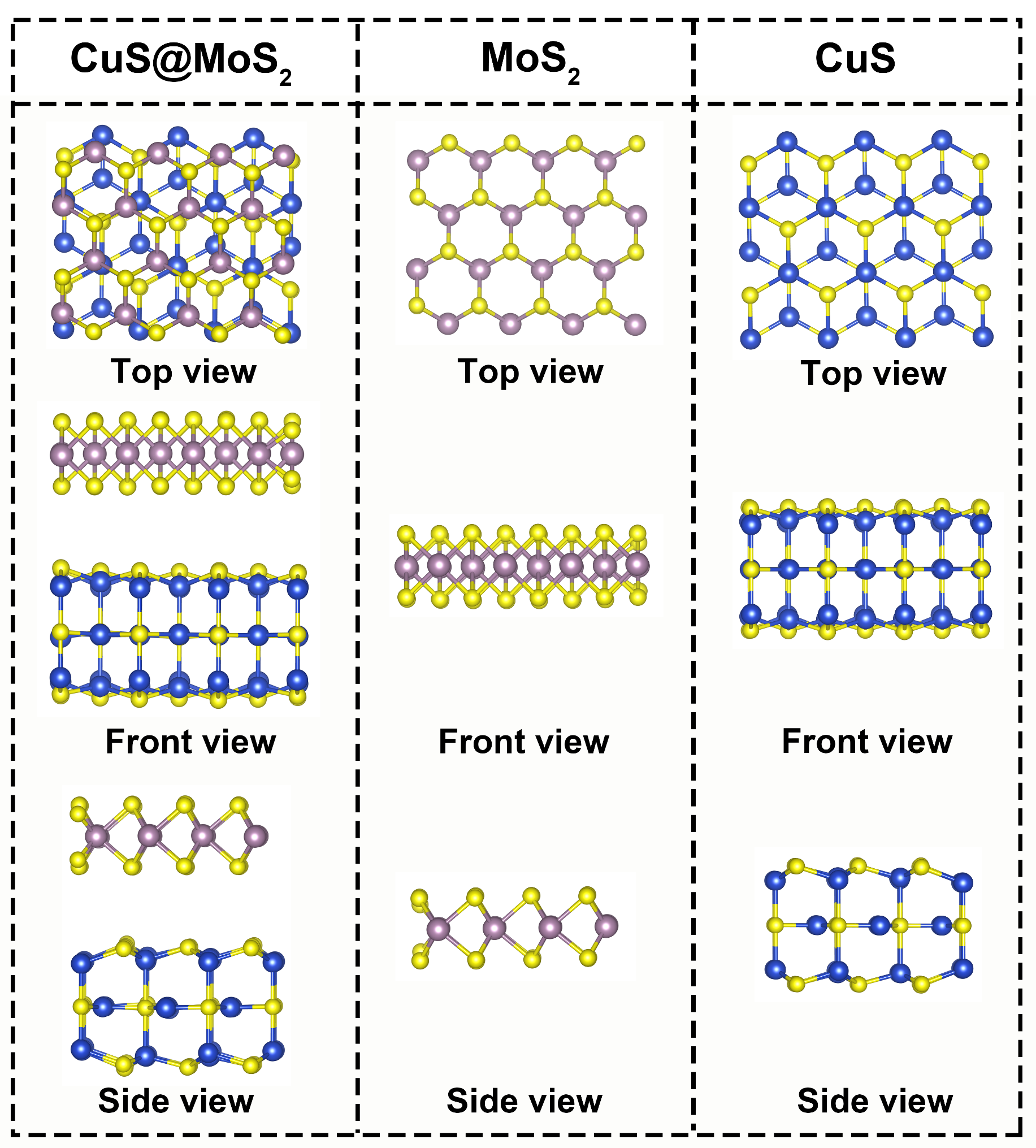


**Figure S13**. Front, side, and top views of the calculated model for CuS@MoS_2_, MoS_2_ and CuS.


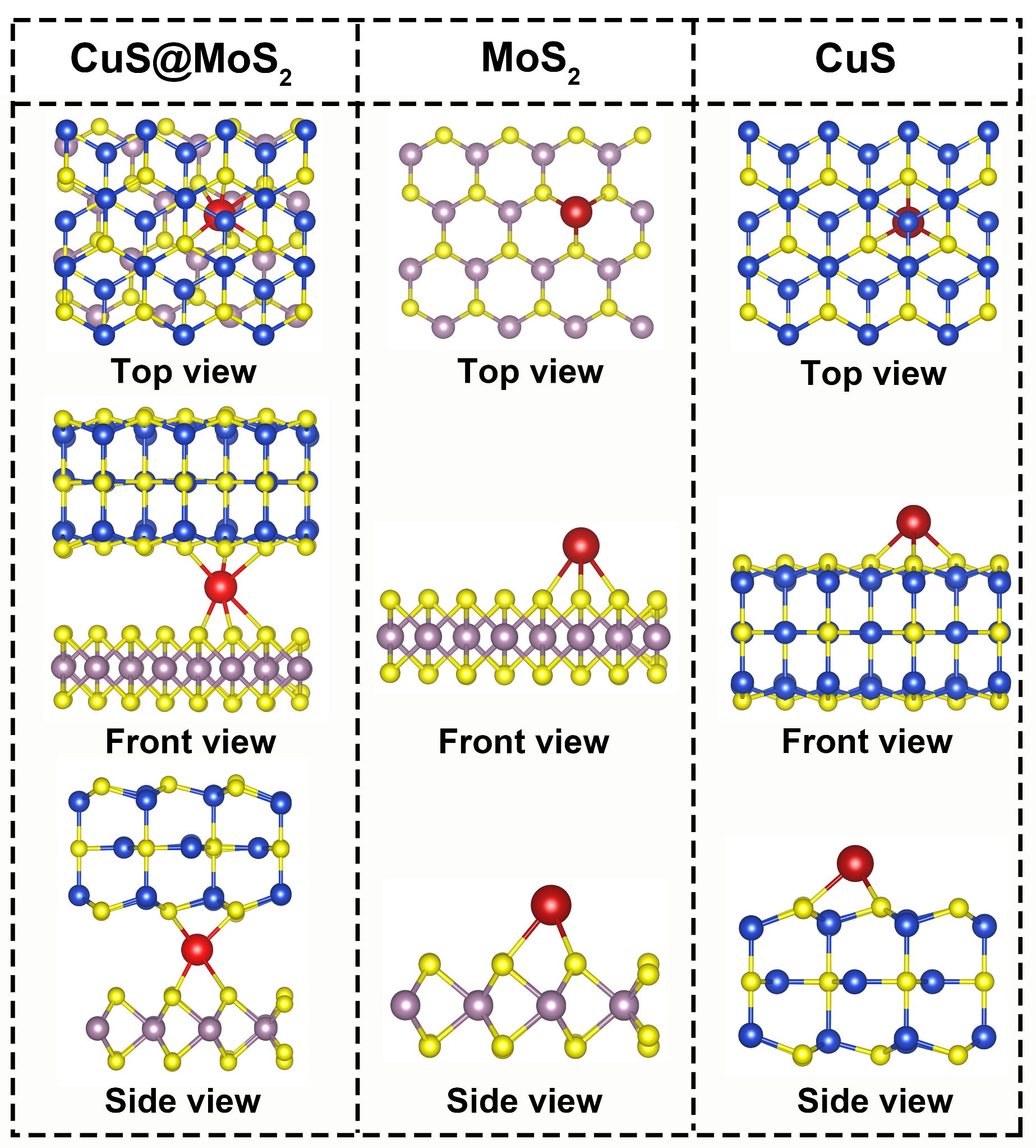


**Figure S14**. Front, side and top views of Na^+^ adsorption structures for CuS@MoS_2_, MoS_2_ and CuS.


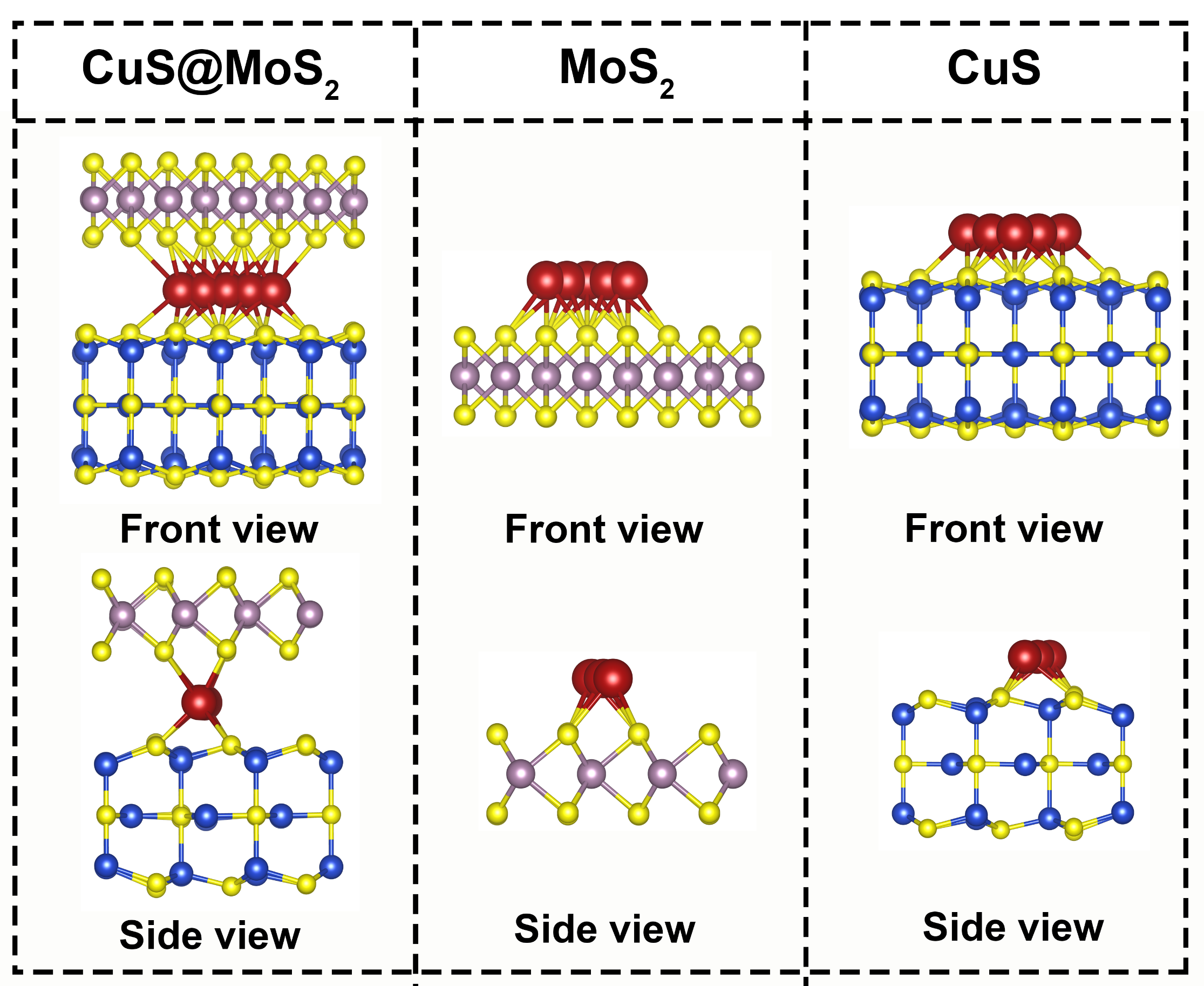


**Figure S15**. Front and side of Schematic diagram of the migration path of Na^+^ in the CuS@MoS_2_, MoS_2_ and CuS interlayers.


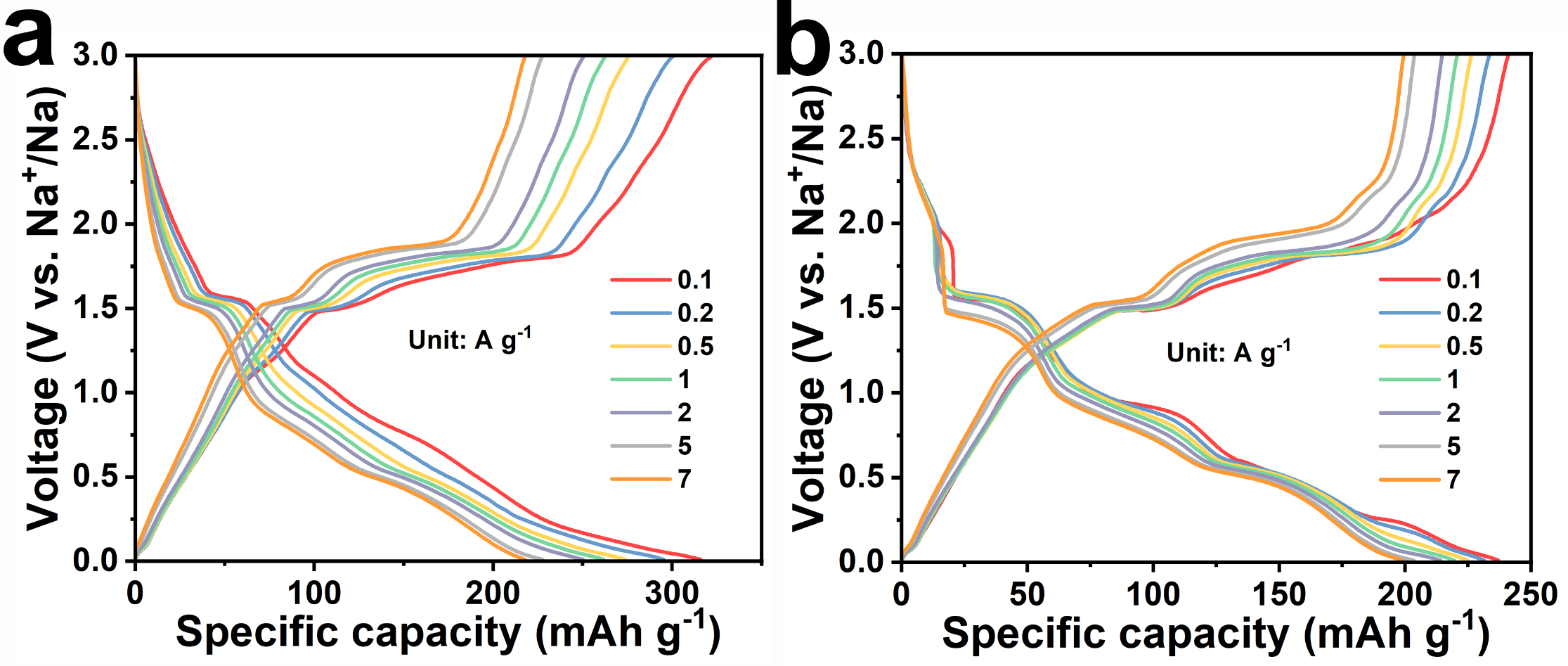


**Figure S16**. Charge-discharge profiles of (a) MoS_2_ and (b) CuS in different current densities.


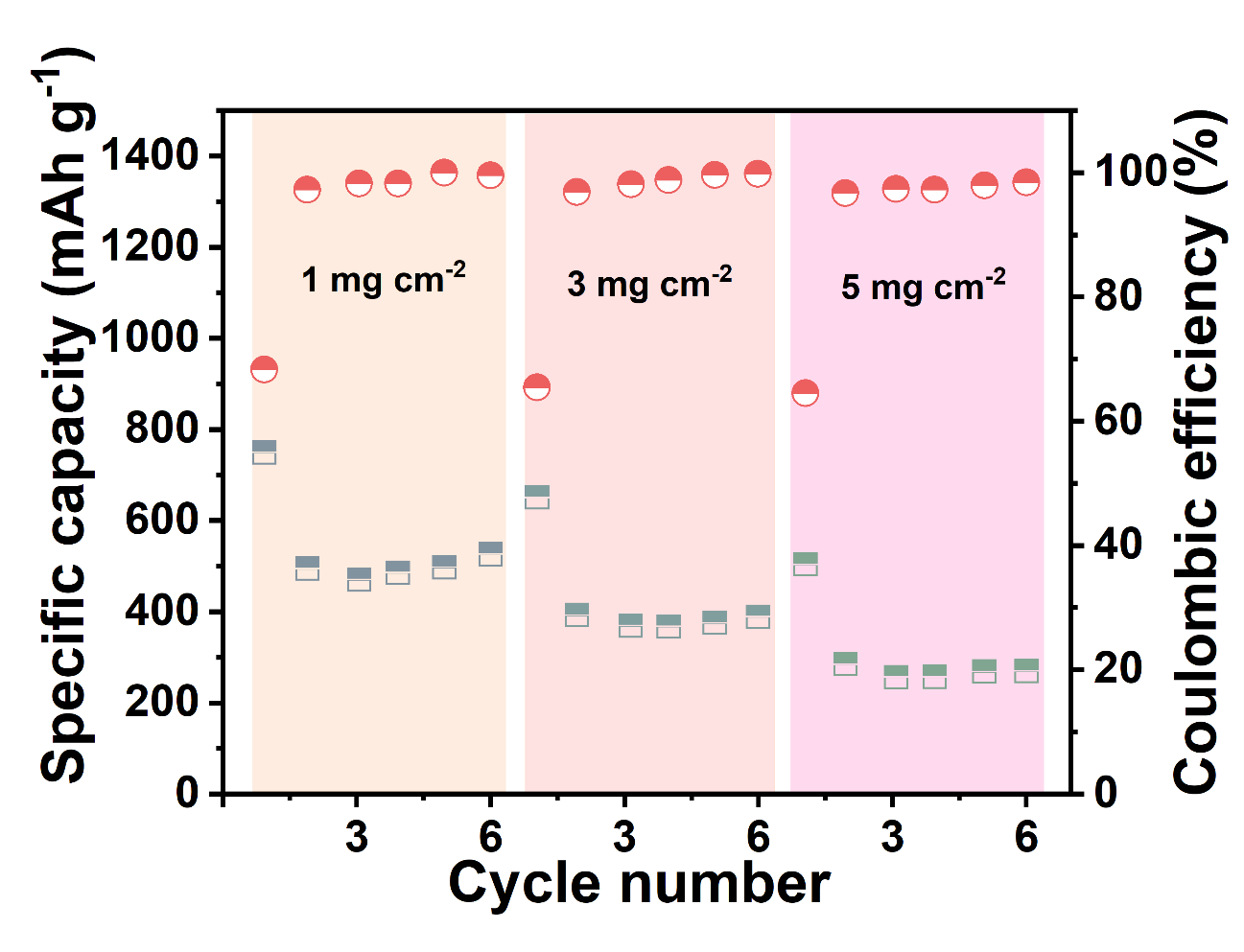


**Figure S17.** Comparison of sodium storage capacity of CuS@MoS_2_ at different loadings.


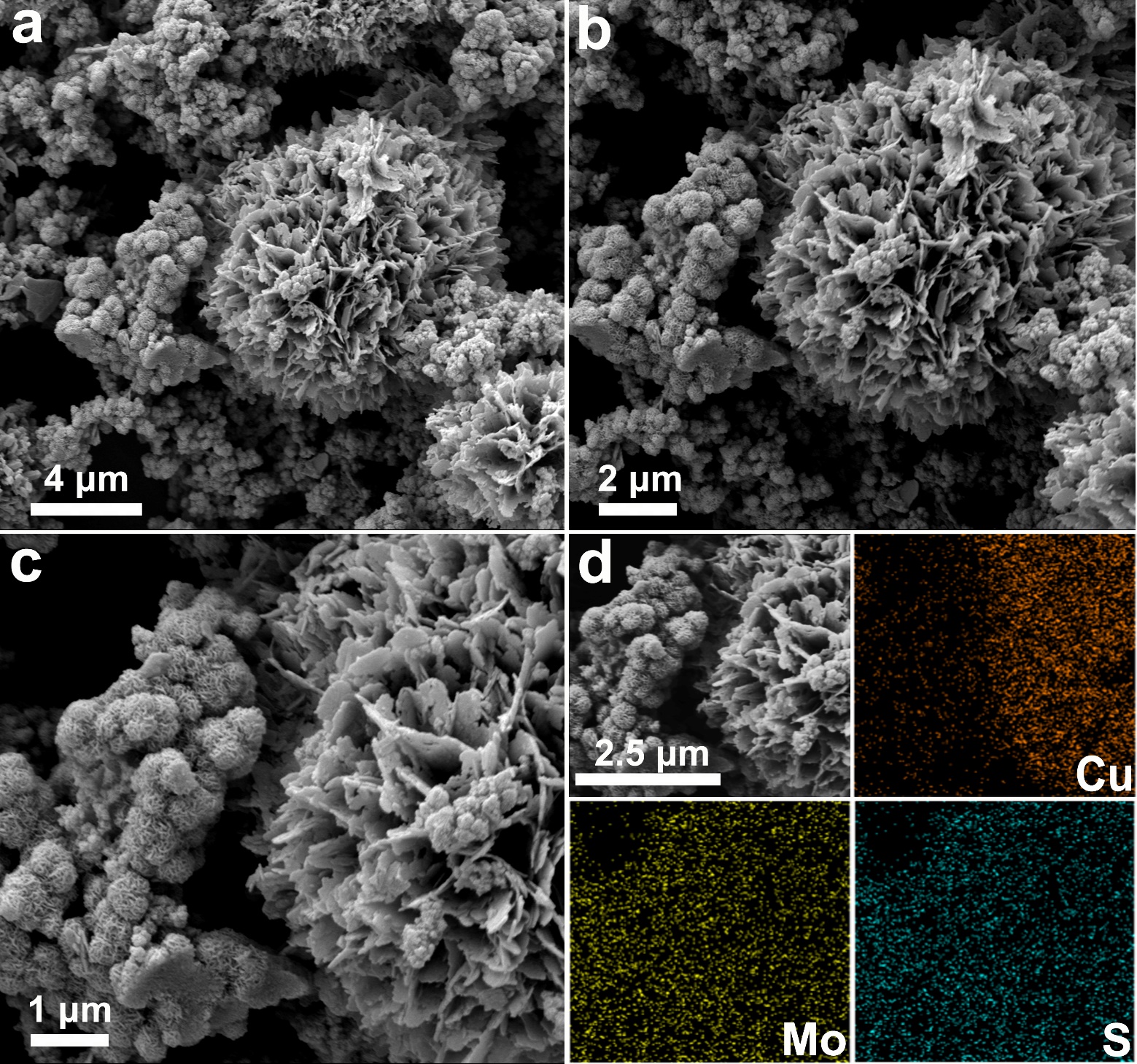


**Figure S18**. (a-c) SEM images and (d) EDS mapping of CuS/MoS_2_.


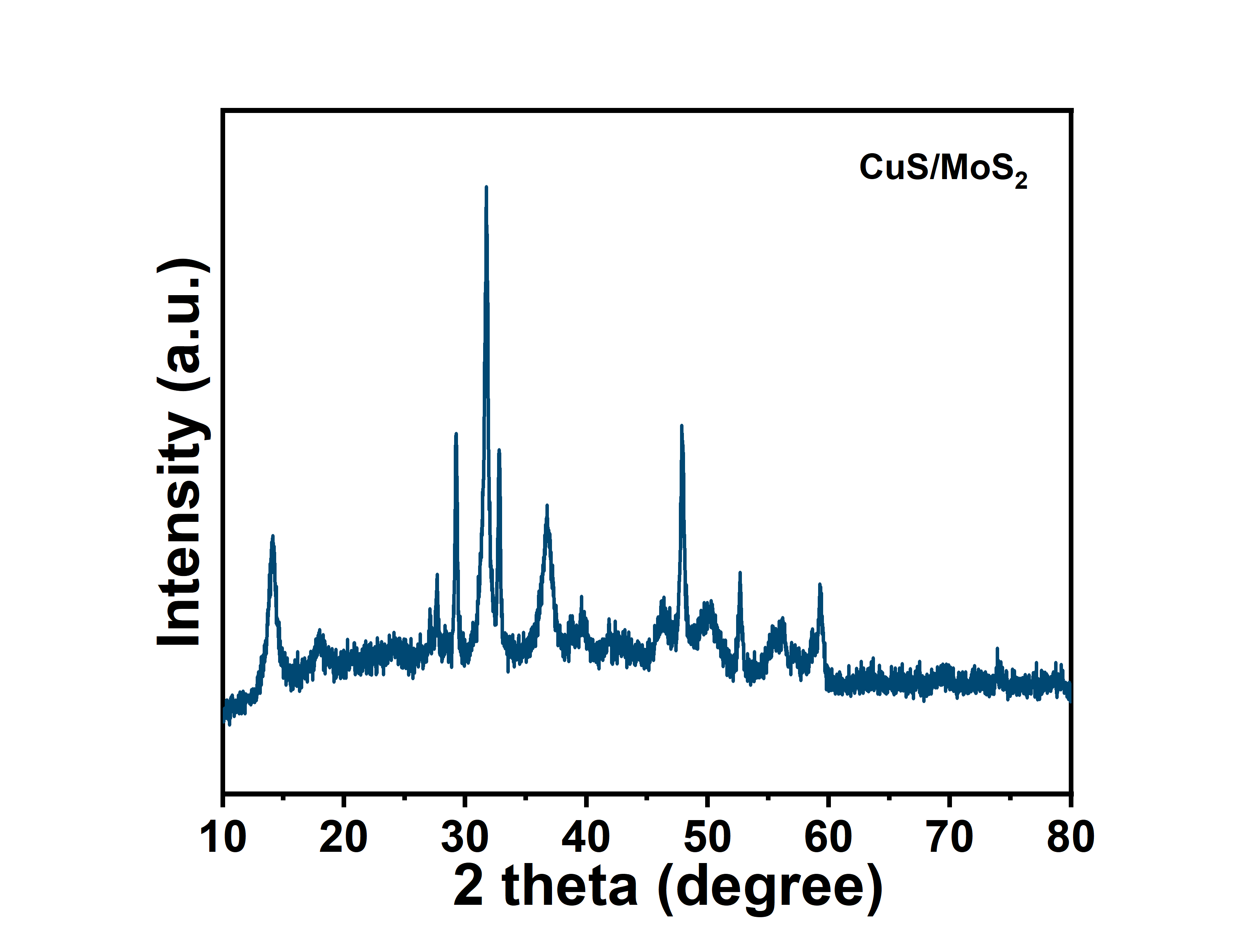


**Figure S19**. XRD pattern of CuS/MoS_2_.


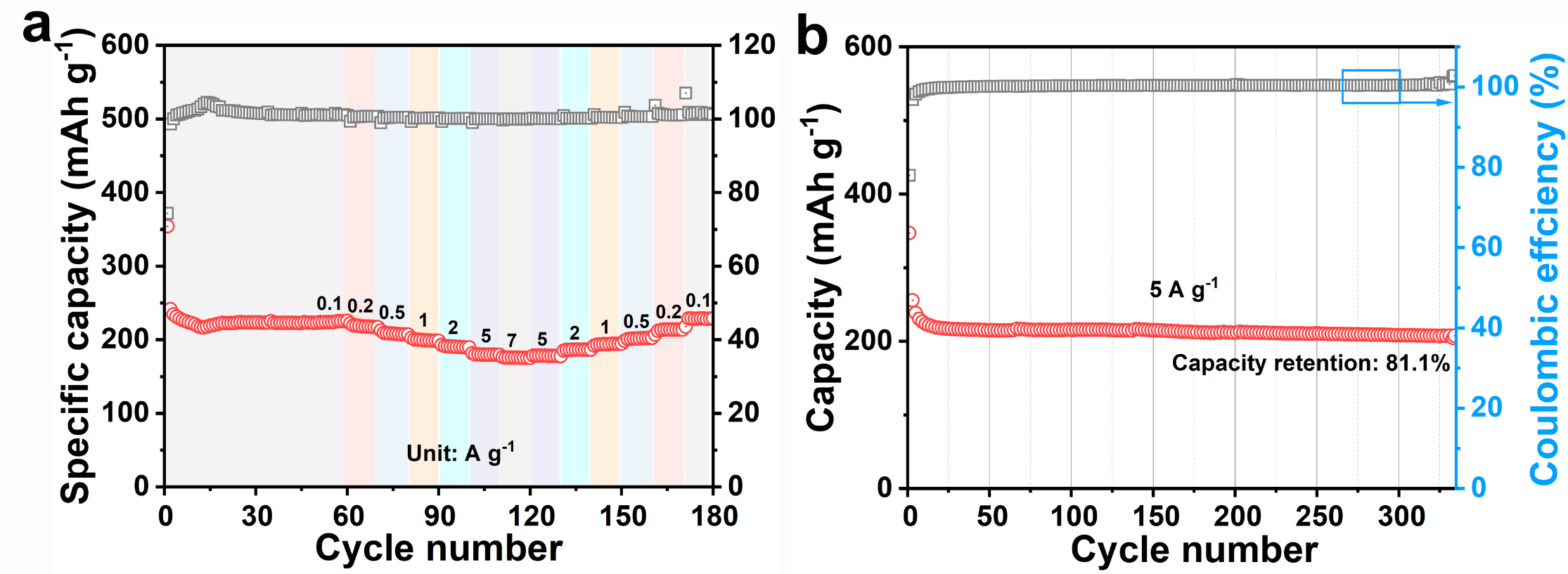


**Figure S20**. (a) Rate capability of CuS/MoS_2_ at various current densities. (b) Cycling performance of CuS/MoS_2_ at 5 A g^-1^.


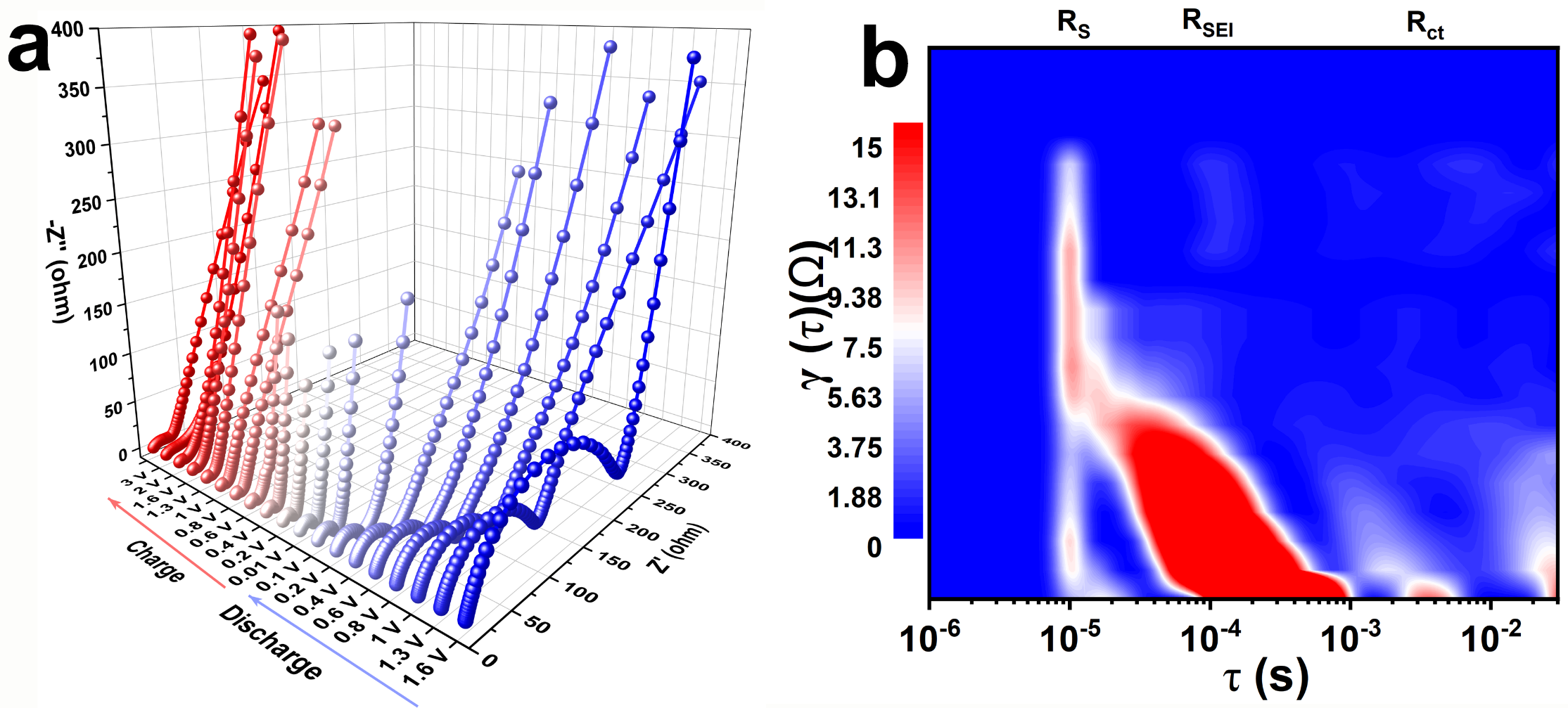


**Figure S21**. (a) In-situ Nyquist plots of MoS_2_ during the initial cycle; (b) The contour plots of DRT for MoS_2_ during the first cycle;


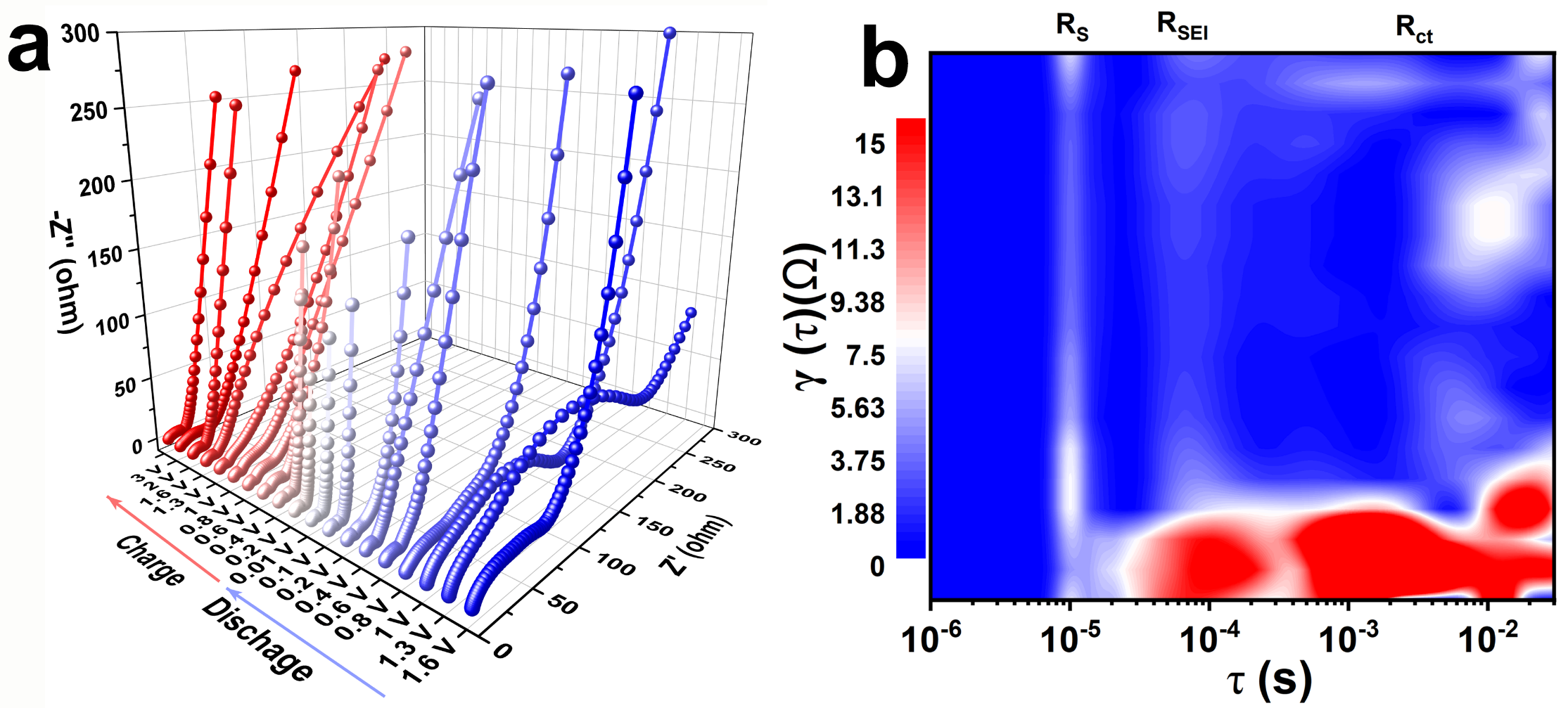


**Figure S22**. (a) In-situ Nyquist plots of CuS during the initial cycle; (b) The contour plots of DRT for CuS during the first cycle;


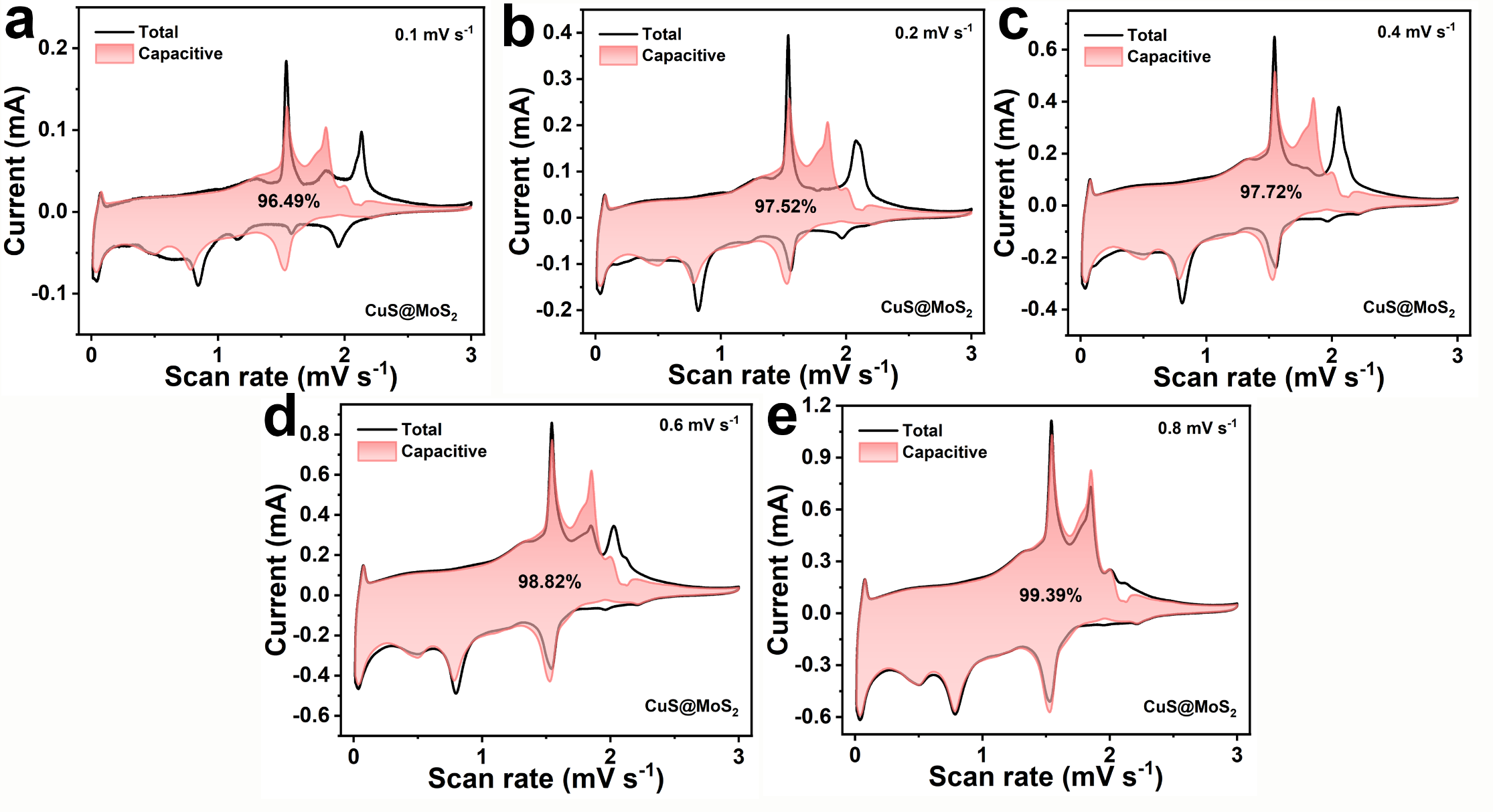


**Figure S23**. Detail capacitive contribution of CuS@MoS_2_ at (a) 0.1 mV s^−1^, (a) 0.2 mV s^−1^, (a) 0.4 mV s^−1^, (a) 0.6 mV s^−1^, (a) 0.8 mV s^−1^.


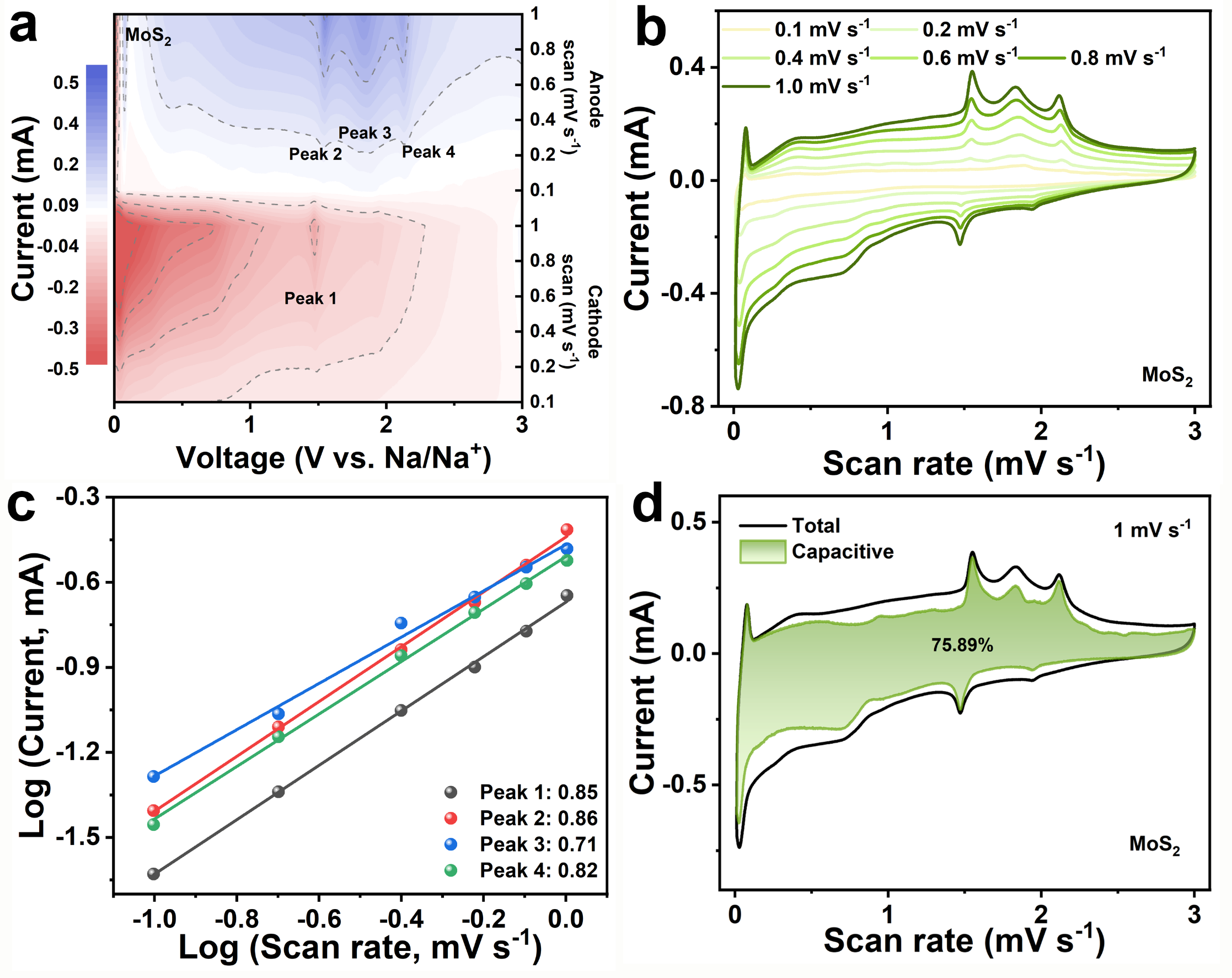


**Figure S24**. (a) Contour plots of CV curves for MoS_2_ at different scan rates; (b) CV curves of MoS_2_ in different Scan rates; (c) Log (i) versus log (v) plots of MoS_2_. (d) Detail capacitive contribution of MoS_2_ at 1.0 mV s^−1^.


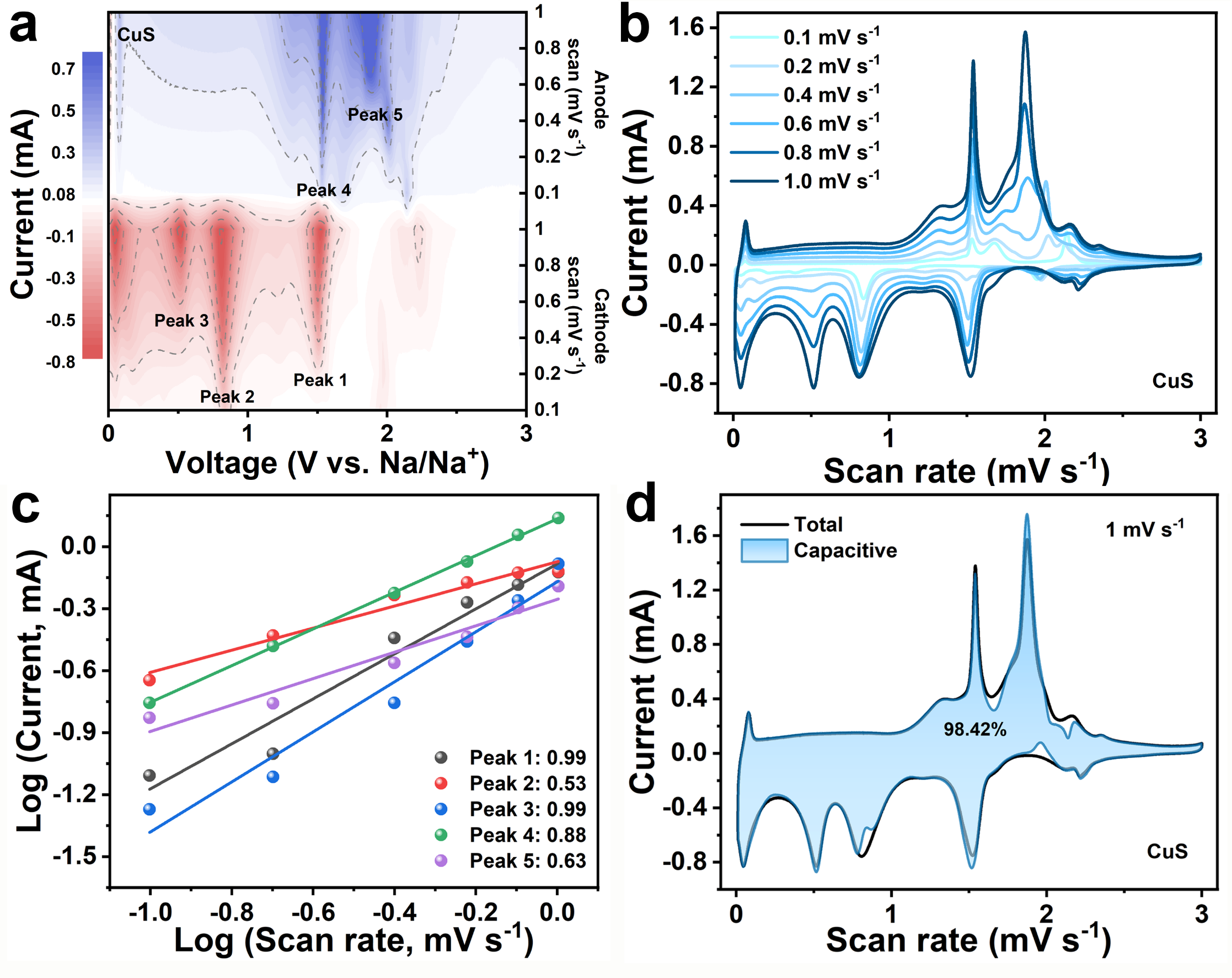


**Figure S25**. (a) Contour plots of CV curves for CuS at different scan rates; (b) CV curves of CuS in different Scan rates; (c) Log (i) versus log (v) plots of CuS. (d) Detail capacitive contribution of CuS at 1.0 mV s^−1^.


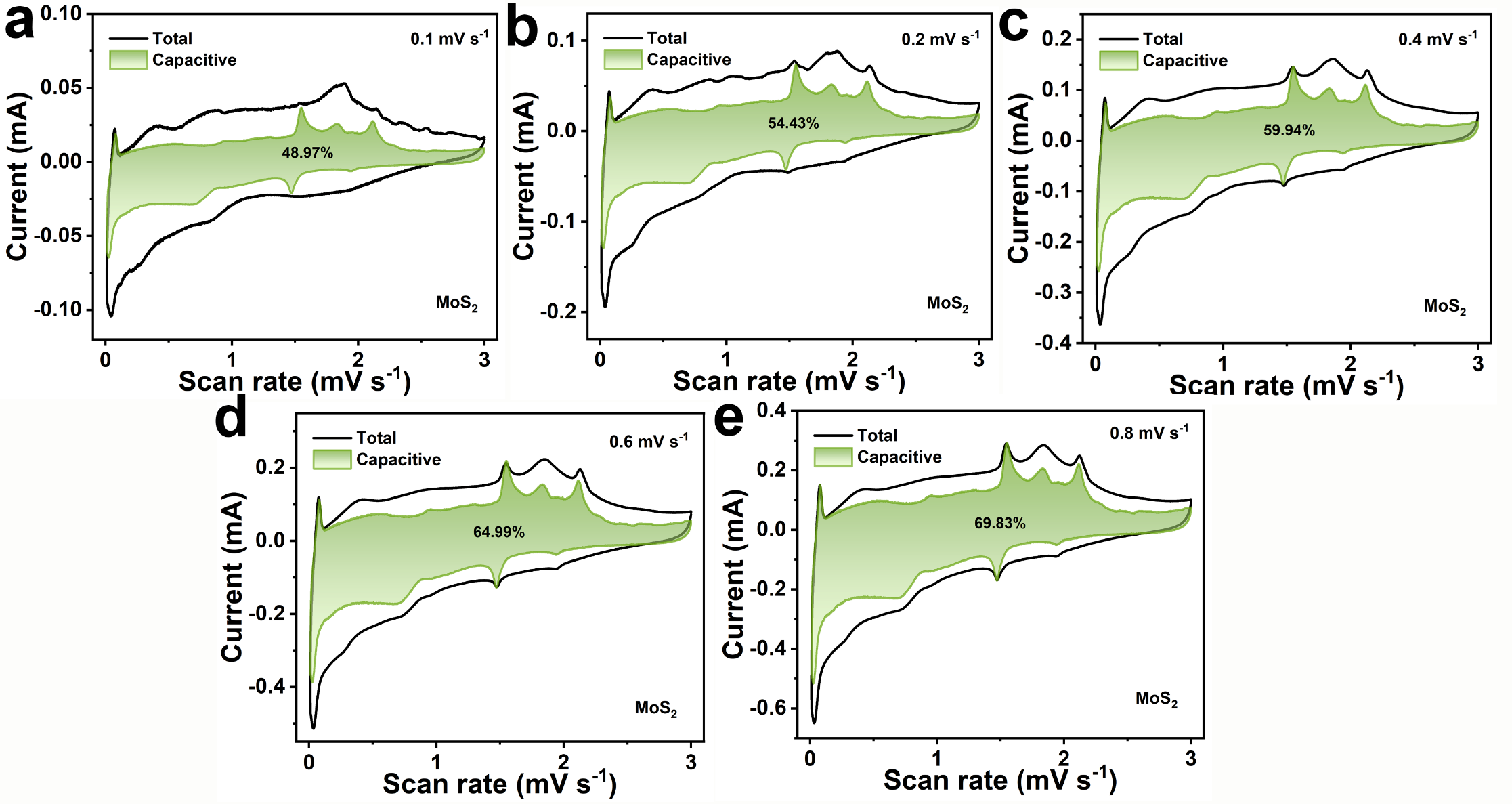


**Figure S26**. Detail capacitive contribution of MoS_2_ at (a) 0.1 mV s^−1^, (b) 0.2 mV s^−1^, (c) 0.4 mV s^−1^, (d) 0.6 mV s^−1^, (e) 0.8 mV s^−1^.


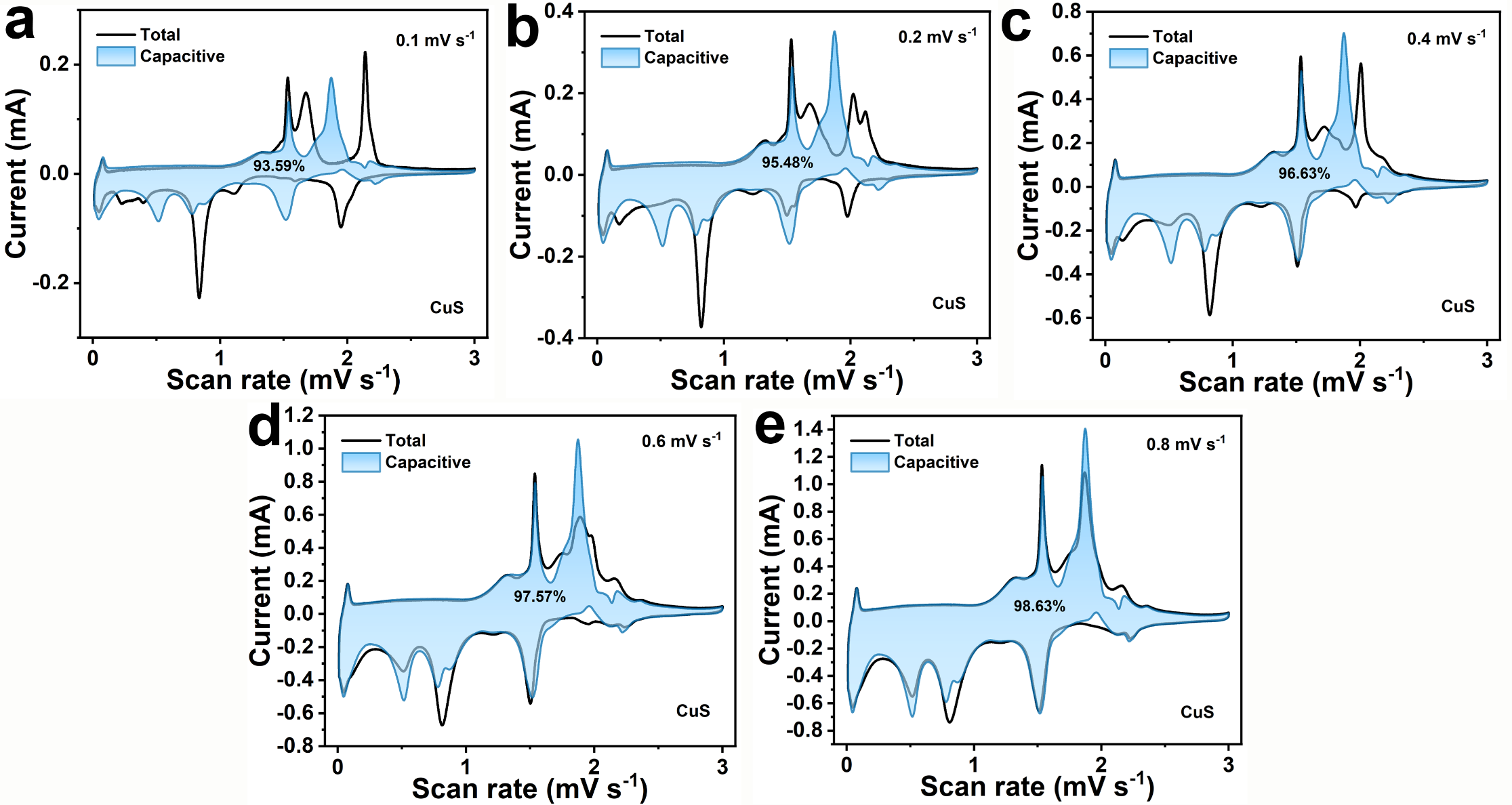


**Figure S27**. Detail capacitive contribution of CuS at (a) 0.1 mV s^−1^, (b) 0.2 mV s^−1^, (c) 0.4 mV s^−1^, (d) 0.6 mV s^−1^, (e) 0.8 mV s^−1^.

**
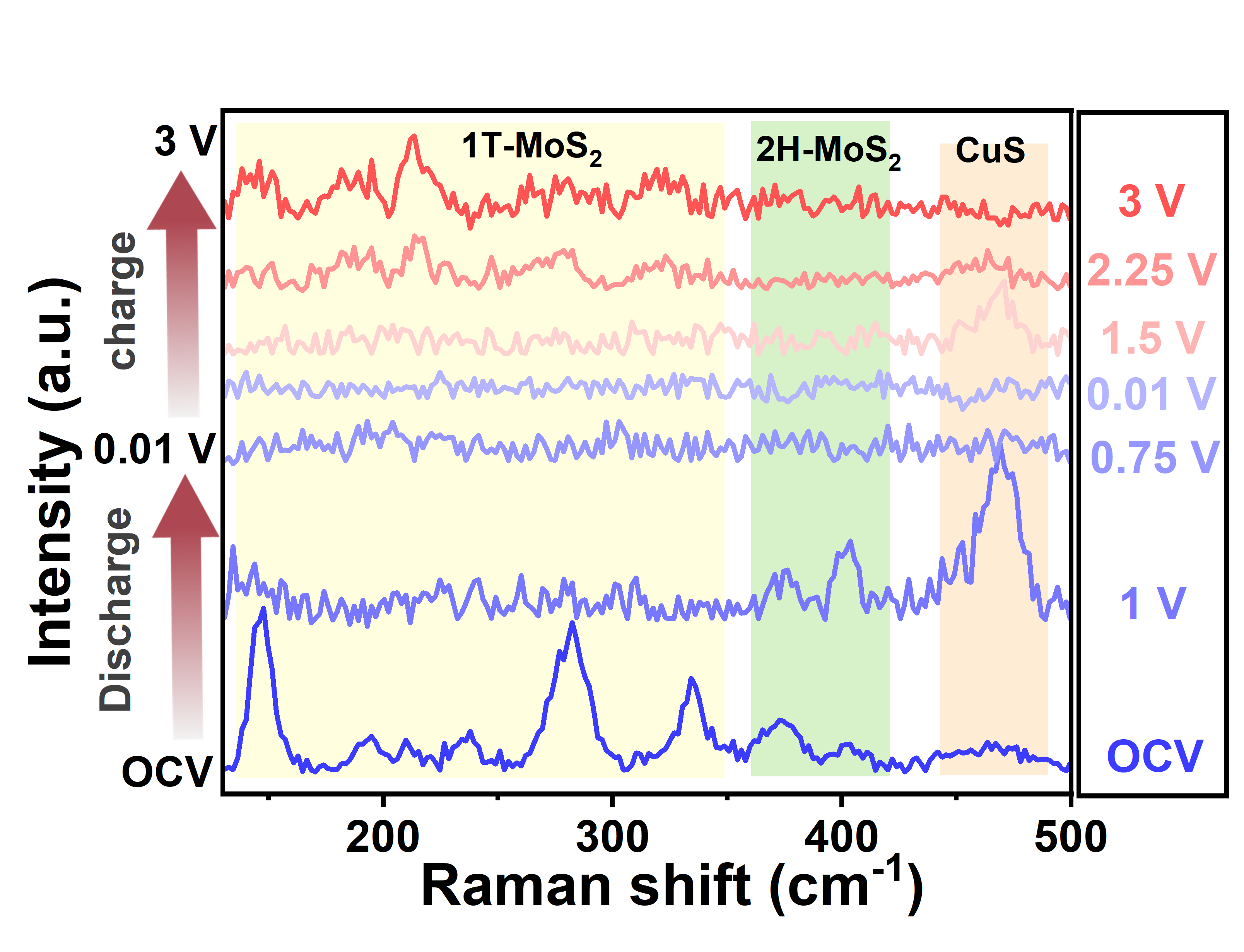
**

**Figure S28**. Stacked patterns of ex-situ Raman spectra of CuS@MoS_2_ during the initial discharge/charge cycle.


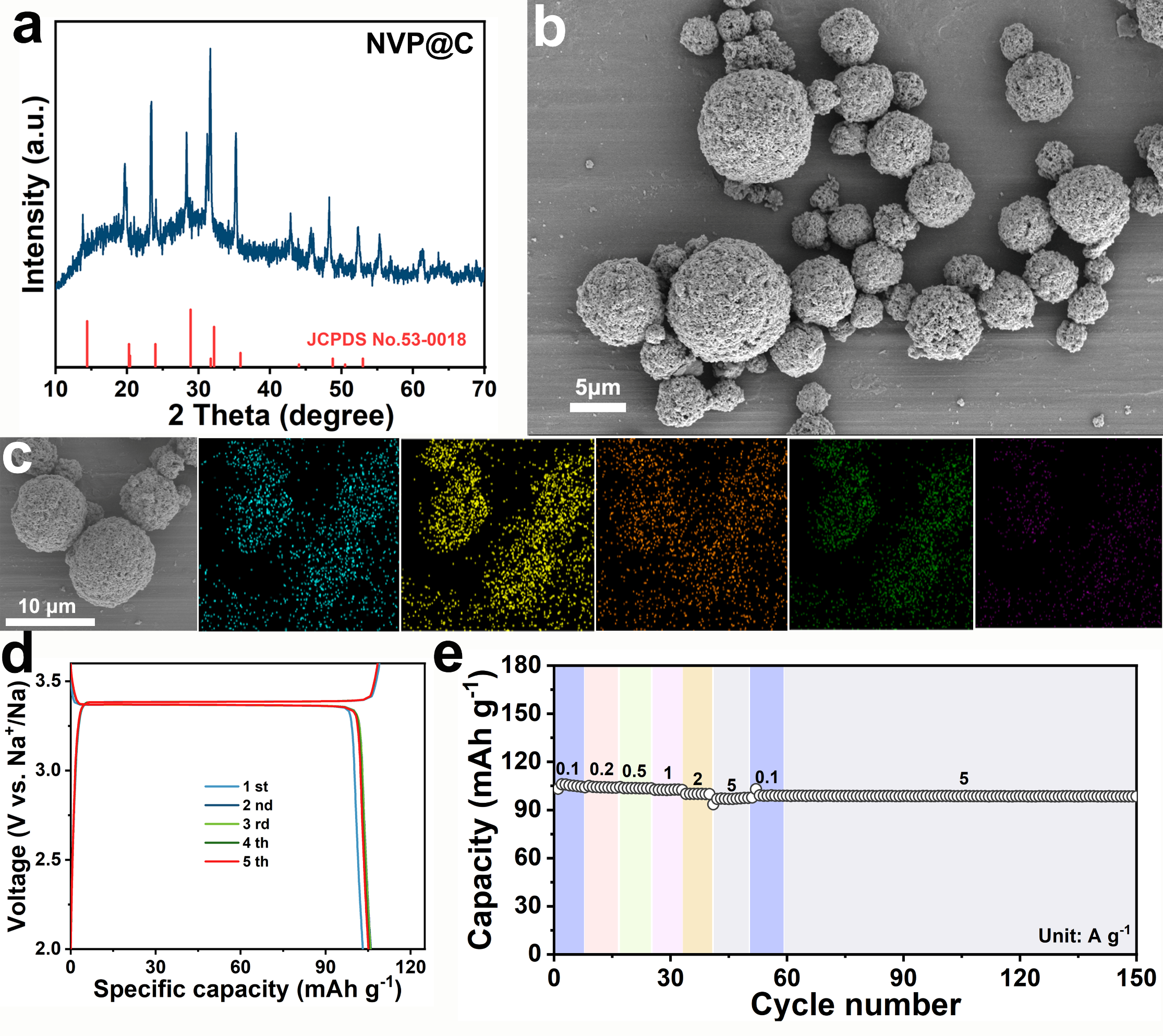


**Figure S29**. (a) XRD pattern and (b) SEM image of Na_3_V_2_(PO_4_)_3_@C. (c) EDS elemental mapping of the Na_3_V_2_(PO_4_)_3_@C. (c) Charge-discharge profiles for the first 5 cycles. (d) Rate capability of the Na_3_V_2_(PO_4_)_3_@C.


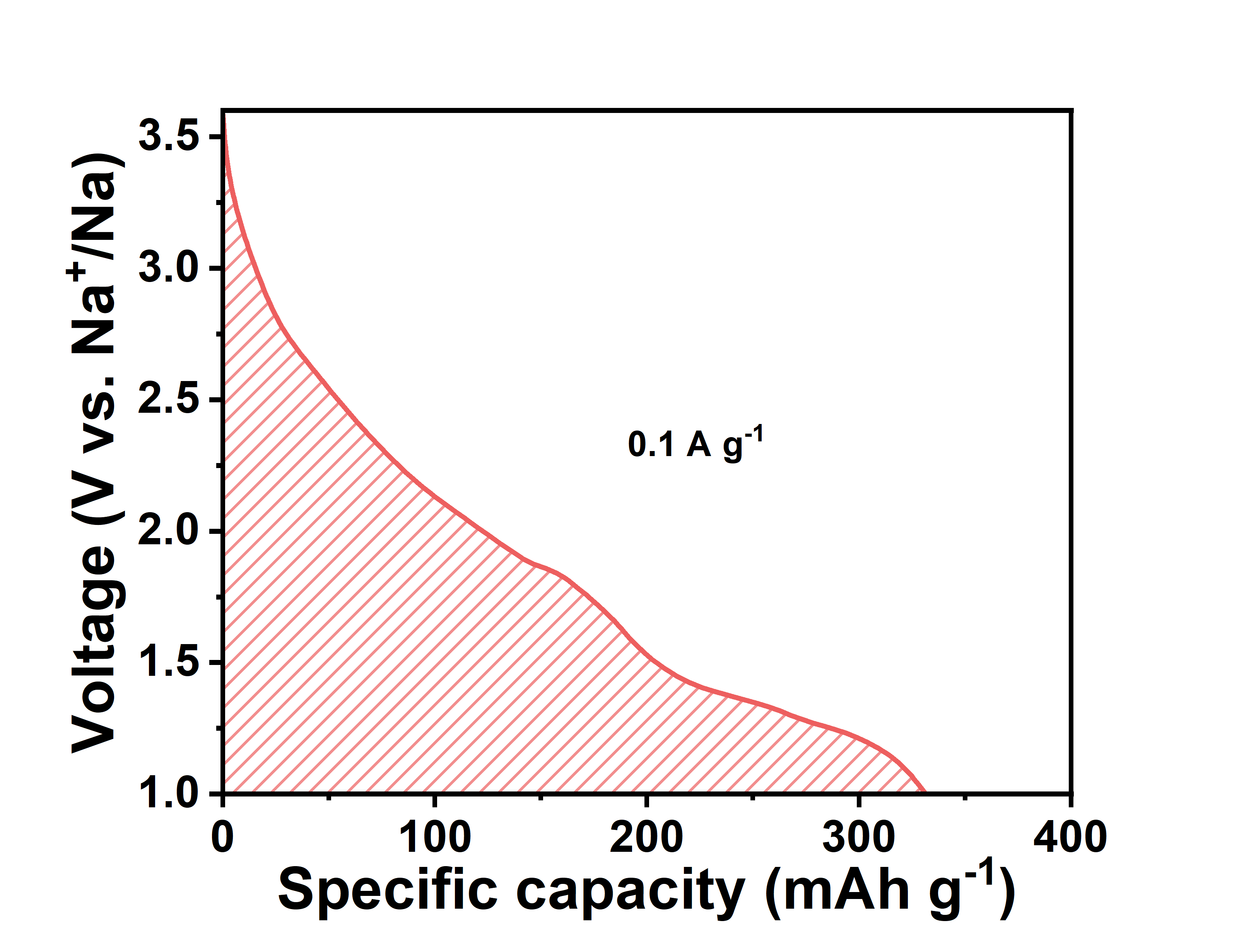


**Figure S30.** Discharge curve and integral area of CuS@MoS_2_//NVP@C SIB at 0.1 A g^-1^.

S_shadow_≈613

V_average_= S_shadow_/Q=613/330.9≈1.85 V


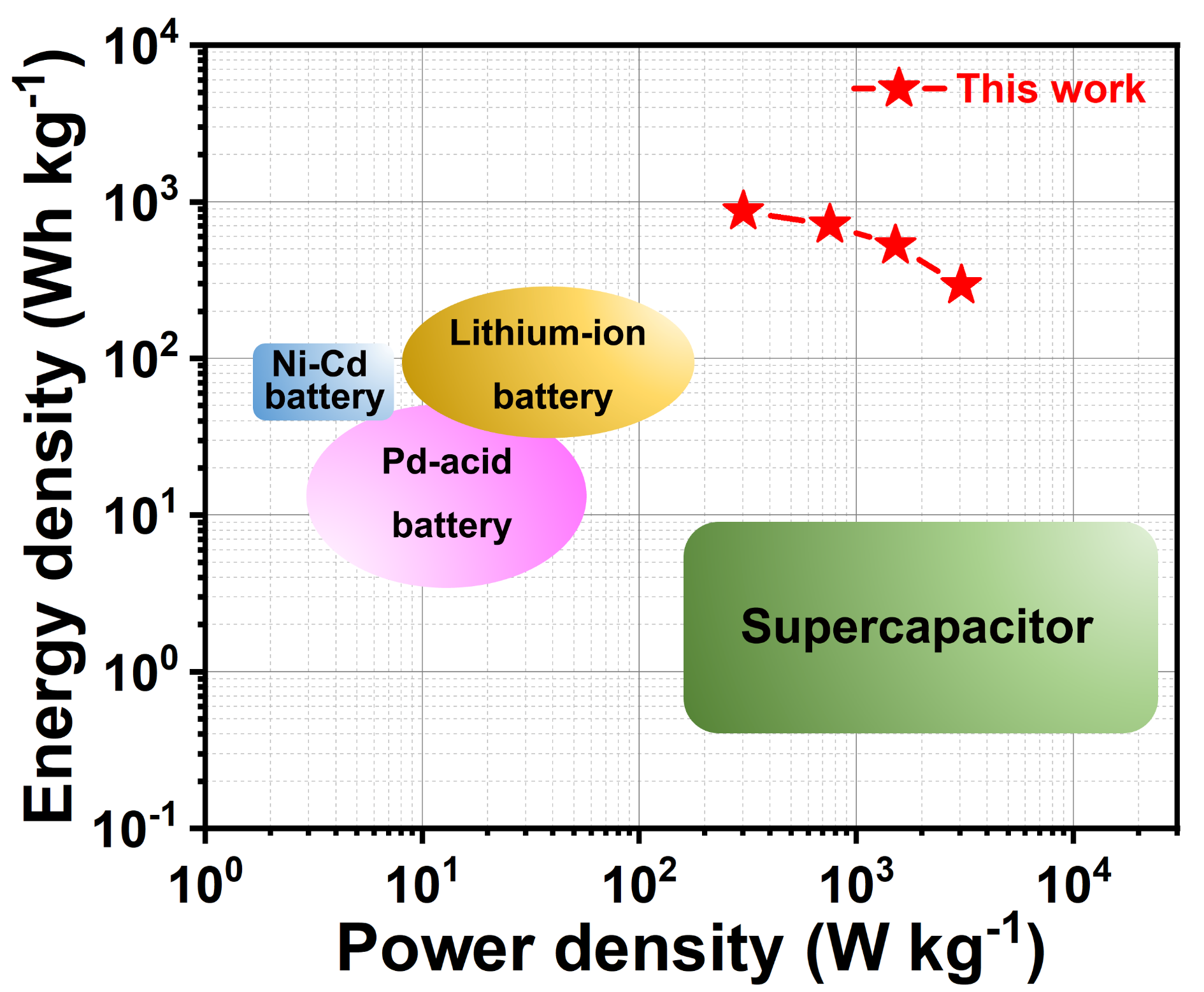


**Figure S31.** Ragone plot of the CuS@MoS_2_//NVP@C SIB and compared it with other energy storage devices.

**Table S1.** The detailed information on XRD refinement (cell parameters, atomic coordinates and occupancy, multiplicity).

| Cell parameters | | | | | | |
| --- | --- | --- | --- | --- | --- | --- |
|  | *a* | *b* | *c* | *α* | *β* | *γ* |
| CuS | 3.789 | 3.789 | 16.338 | 90.000 | 90.000 | 120.000 |
| MoS_2_ | 3.298 | 3.298 | 18.831 | 90.000 | 90.000 | 120.000 |
| Atomic coordinates and occupancy, multiplicity | | | | | | |
|  | Name | *x* | *y* | *z* | *occ.* | *Mult.* |
| CuS | Cu1 | 0.33333(0) | 0.66667(0) | 0.75000(0) | 0.083(0) | 2 |
|  | Cu2 | 0.33333(0) | 0.66667(0) | 0.10497(0) | 0.167(0) | 4 |
|  | S1 | 0.33333(0) | 0.66667(0) | 0.25000(0) | 0.083(0) | 2 |
|  | S2 | 0.00000(0) | 0.00000(0) | 0.06342(0) | 0.167(0) | 4 |
| MoS_2_ | Mo1 | 0.00000(0) | 0.00000(0) | -0.00462(0) | 0.167(0) | 3 |
|  | S1 | 0.00000(0) | 0.00000(0) | 0.25028(0) | 0.167(0) | 3 |
|  | S2 | 0.00000(0) | 0.00000(0) | 0.42907(0) | 0.167(0) | 3 |

**Table S2.** EXAFS fitting parameters at the Cu K-edge for various samples（*Ѕ*_0_^2^=0.72 from Cu-foil）

|  | shell | CN*^a^* | R*^b^*(Å) | σ^2^*^c^*(Å^2^) | ΔE_0_*^d^*(eV) | R factor |
| --- | --- | --- | --- | --- | --- | --- |
| Cu-foil | Cu-Cu | 12 | 2.54±0.01 | 0.0086 | 3.8±0.6 | 0.0030 |
| Cu-1 | Cu-S | 3.8±0.3 | 2.26±0.01 | 0.0084 | 1.6±1.0 | 0.0165 |
| Cu-2 | Cu-S | 3.9±0.4 | 2.26±0.01 | 0.0088 | 3.8±1.1 | 0.0174 |

*^a^CN*: coordination numbers; *^b^R*: bond distance; *^c^σ*^2^: Debye-Waller factors; *^d^* Δ*E*_0_: the inner potential correction. R factor: goodness of fit.

The obtained XAFS data was processed in Athena (version 0.9.26) [J. Synchrotron Rad. 2005, 12, 537] for background, pre-edge line and post-edge line calibrations. Then Fourier transformed fitting was carried out in Artemis (version 0.9.26) [J. Synchrotron Rad. 2005, 12, 537]. The k^3^ weighting, k-range of 3–~12 Å^-1^and R range of 1-3 Å were used for the fitting of Cu-foil; The k^3^ weighting, k-range of 3 –~12 Å^-1^and R range of 1–3 Å were used for the fitting of Cu-Sample.

**Table S3.** Bader charge calculation results.

|  | Mo | Cu | S |
| --- | --- | --- | --- |
| MoS_2_ | 1.166 | - | -0.583 |
| CuS | - | 0.429 | -0.429 |
| CuS@MoS_2_ | 1.136 | 0.495 | -0.531 |

The numbers in the table represent the average charge of the atom (positive values are lost electrons, negative values are gained electrons).

**Table S4.** The comparisons of electrochemical performance for SIBs half cells between this work and previously transition metal chalcogenides electrode materials.

| Materials | Current density  (A g^-1^) | Cycling number | Specific capacity  (mAh g^-1^) | Refs |
| --- | --- | --- | --- | --- |
| CuS@MoS_2_ | **5** | **3200** | **560** | **This work** |
| Cu_2_Se@C | 1 | 200 | 207 | [1] |
| Fe_7_Se_8_@C | 3 | 550 | 219 | [1] |
| NiSe@C | 3 | 2000 | 160 | [1] |
| SMSC-1-Mo-2 | 5 | 2500 | 310 | [2] |
| ZnS@NC | 0.1 | 1000 | 450 | [3] |
| H-WS_2_ | 5 | 140 | 193.2 | [4] |
| CuS@CoS_2_ | 0.5 | 500 | 410 | [5] |
| CuS@carbon@MoS_2_ | 0.3 | 200 | 300 | [6] |
| Ti_3_C_2_T_x_/CuS | 3 | 800 | 347 | [7] |
| MoS_2_@CoS_2_ | 5 | 550 | 491 | [8] |
| MnS-MoS_2_ | 1 | 500 | 214 | [9] |
| Cu_1.96_S@NC | 5 | 2000 | 375.6 | [10] |

**Table S5.** The comparisons of electrochemical performance for SIBs full cells between this work and previous transitions metal sulfide-based full cells.

| SIBs | Current density  (A g^-1^) | Cycling number | Specific capacity  (mAh g^-1^) | Refs |
| --- | --- | --- | --- | --- |
| CSC//Na_1.5_VPO_4.8_F_0.7_ | 0.25 | 120 | 60 | [11] |
| NNMO-WZSC | 1 | 500 | 100 | [12] |
| GeS_2_//Na_3_V_2_(PO_4_)_2_O_2_F | 0.1 | 100 | 85 | [13] |
| Fe-Bi_2_S_3_/C//NVP | 1 | 90 | 308 | [14] |
| Cu_2-x_Se@NC//NVP | 0.5 | 200 | 161 | [15] |
| NHCFs-S-Fe_7_S_8_//NVP@C | 1.1 | 120 | 80 | [16] |
| CuSe/ZnSe@NC//NVP | 0.5 | 120 | 153 | [17] |
| NVP@rGO//Cu_2_Se | 0.1 | 90 | 183 | [18] |
| SRNDC-700//NVP | 1 | 100 | 73 | [19] |
| Cu_2−x_Se@C//NVP/C | 0.6 | 500 | 85 | [20] |
| CuS@MoS_2_//NVP@C | **1** | **600** | **186.55** | **This work** |

**References**

[1] X. Xu, J. Liu, J. Liu, L. Ouyang, R. Hu, H. Wang, L. Yang, M. Zhu, *Adv. Funct. Mater.* **2018**, *28*, 1707573.

[2] X. Zhang, W. Weng, H. Gu, Z. Hong, W. Xiao, F. Wang, W. Li, D. Gu, *Adv. Mater.* **2021**, *34*, 2104427.

[3] K. Yang, H. Fu, Y. Duan, M. Wang, M. X. Tran, J. K. Lee, W. Yang, G. Liu, *Energy Environ. Mater.* **2022**, *6*, e12380.

[4] X. Luo, J. Huang, Y. Huang, L. Cao, J. Li, Y. Wang, Z. Xu, S. Wei, K. Kajiyoshi, *J. Mater. Chem. A* **2021**, *9*, 21366-21378.

[5] Y. Fang, B. Y. Guan, D. Luan, X. W. Lou, *Angew. Chem. Inter. Edit.* **2019**, *58*, 7739-7743.

[6] Y. Fang, D. Luan, Y. Chen, S. Gao, X. W. Lou, *Angew. Chem. Inter. Edit.* **2020**, *59*, 7178-7183.

[7] P. Huang, H. Ying, S. Zhang, Z. Zhang, W.-Q. Han, *J. Mater. Chem. A* **2022**, *10*, 22135-22144.

[8] S. Gao, Y. He, H. Li, G. Yue, Z. Cui, Y. Li, J. Bai, N. Wang, Q. Zhang, Y. Yu, Y. Zhao, *Energy Storage Mater.* **2024**, *65*, 103170.

[9] F. Chen, D. Shi, M. Yang, H. Jiang, Y. Shao, S. Wang, B. Zhang, J. Shen, Y. Wu, X. Hao, *Adv. Funct. Mater.* **2020**, *31*, 2007132.

[10] H. Peng, W. Miao, S. Cui, Z. Liu, B. Tao, W. Hou, G. Ma, Z. Lei, *Small* **2024**, *20*, 2404957.

[11] Y. Tang, Y. Wei, A. F. Hollenkamp, M. Musameh, A. Seeber, T. Jin, X. Pan, H. Zhang, Y. Hou, Z. Zhao, X. Hao, J. Qiu, C. Zhi, *Nano-Micro Lett.* **2021**, *13*, 178.

[12] Y. Li, J. Qian, M. Zhang, S. Wang, Z. Wang, M. Li, Y. Bai, Q. An, H. Xu, F. Wu, L. Mai, C. Wu, *Adv. Mater.* **2020**, *32*, 2005802.

[13] C. C. Li, B. Wang, D. Chen, L.-Y. Gan, Y. Feng, Y. Zhang, Y. Yang, H. Geng, X. Rui, Y. Yu, *ACS Nano* **2019**, *14*, 531-540.

[14] H. Yuan, F. Ma, X. Wei, S. Jia, P. Kang, Y. Yu, X. Yang, J.-L. Lan, *Mate. Today Energy* **2022**, *28*, 101084.

[15] H. Peng, W. Miao, S. Cui, Z. Liu, X. Wang, B. Tao, W. Hou, Z. Zhang, G. Ma, *Chem. Eng. J.* **2024**, *487*, 150701.

[16] F. Wang, Z. Liu, H. Feng, Y. Wang, C. Zhang, Z. Quan, L. Xue, Z. Wang, S. Feng, C. Ye, J. Tan, J. Liu, *Small* **2023**, *19*, 2302200.

[17] X. Xie, X. Ma, Z. Yin, H. Tong, H. Jiang, Z. Ding, L. Zhou, *Chem. Eng. J.* **2022**, *446*.

[18] L. Shao, S. Wang, J. Qi, Z. Sun, X. Shi, Y. Shi, X. Lu, *Mater. Today Phys.* **2021**, *19*, 100422.

[19] B. Yin, S. Liang, D. Yu, B. Cheng, I. L. Egun, J. Lin, X. Xie, H. Shao, H. He, A. Pan, *Adv. Mater.* **2021**, *33*, 2100808.

[20] H. Li, H. Zhang, M. Zarrabeitia, H. P. Liang, D. Geiger, U. Kaiser, A. Varzi, S. Passerini, *Adv. Sustain. Syst.* **2022**, *6*, 2200109.
